# Detachable DNA Assembly Module to Dissect Tumor Cells Heterogeneity via RNA Pinpoint Screening

**DOI:** 10.1002/advs.202401253

**Published:** 2024-10-18

**Authors:** Wei Liu, Ni Liao, Yanmei Lei, Wenbin Liang, Xia Yang, Ruo Yuan, Chaoyong Yang, Ying Zhuo

**Affiliations:** ^1^ Key Laboratory of Luminescence Analysis and Molecular Sensing (Southwest University) Ministry of Education College of Chemistry and Chemical Engineering Southwest University Chongqing 400715 P. R. China; ^2^ College of Biological and Chemical Engineering Panzhihua University Panzhihua 617000 P. R. China; ^3^ Institute of Molecular Medicine Renji Hospital School of Medicine Shanghai Jiao Tong University Shanghai 200127 P. R. China; ^4^ The MOE Key Laboratory of Spectrochemical Analysis and Instrumentation Department of Chemical Biology College of Chemistry and Chemical Engineering Xiamen University Xiamen 361005 P. R. China

**Keywords:** cells discrimination, DNA assembly module, proportion evaluation, RNA screening

## Abstract

Differential RNA expression is becoming increasingly valuable in evaluating tumor heterogeneity for a better understanding of malignant tumors and guiding personalized therapy. However, traditional techniques for analyzing cellular RNA are mainly focused on determining the absolute level of RNA, which may lead to inaccuracies in understanding tumor heterogeneity, primarily due to i) the subtle differences in certain RNA types that have similar total concentrations and ii) the existence of variations in RNA expression across different samples. Herein, a detachable DNA assembly module is proposed that is capable not only of quantifying the expression level of target RNA but also of innovatively evaluating its proportion within its RNA family population through a sequential assembly and disassembly route. Using the let‐7 family as an experimental model, a significant difference is discovered in let‐7a proportion between normal mammary epithelial cells and breast cancer cells, a characteristic that is often missed in bulk analysis of traditional techniques. By combining concentration and proportion information, the detachable DNA assembly module demonstrates markedly higher efficiency in discerning among various types of cells compared to traditional techniques. This innovative assembly module is expected to offer a new perspective to highlight tumor heterogeneity and guide personalized therapy.

## Graphical abstract

A DNA module with the disassembly property demonstrates the ability to quantify an individual target RNA in its highly homologous RNA family and determines its proportion within the family. The DNA module has been shown to accurately differentiate between multiple cell types, facilitating the identification of heterogeneity within tumor cells.

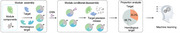

## 1 Introduction

Personalized therapy, a medical model that formulates targeted treatment programs based on personal genome information and internal environment information, is critical for improving the five‐year survival rates of tumor patients.^[^ ^1^, ^2^ ^]^ At present, tumor heterogeneity is manifested in multiple aspects, ranging from cellular genotypes to cellular phenotypes among individual patients or between patients, wherein genomic heterogeneity assessment as a diagnostic basis dominates the regimen formulation of personalized therapy.^[^ ^3^, ^4^ ^]^ Among them, RNA fulfills essential functions of transcription, translation, and regulation in both upstream and downstream gene expression pathways.^[^ ^5^, ^6^ ^]^ In particular, the abnormal expression of cellular RNA has been closely associated with the occurrence, development, and prognosis of tumors.^[^ ^7^, ^8^, ^9^, ^10^, ^11^ ^]^ Therefore, the reliable differential evaluation of RNA expression is highly informative for timely tumor diagnosis and personalized therapy.

Despite single‐cell sequencing being widely applied in RNA expression investigation, in situ analysis within cells still encounters difficulties because of its strict experimental conditions and cumbersome operational procedures.^[^ ^12^, ^13^, ^14^ ^]^ In recent years, molecular imaging has been demonstrated as a feasible technical route to monitor RNA visually with spatiotemporal resolution.^[^ ^15^, ^16^, ^17^, ^18^, ^19^ ^]^ However, existing molecular techniques for analyzing cellular RNA primarily rely on the absolute level of RNA.^[^ ^20^, ^21^ ^]^ These techniques may lead to inaccuracies in understanding tumor heterogeneity, primarily due to i) the subtle differences in certain RNA types that have similar total concentrations and ii) the existence of variations in RNA expression across different samples. Moreover, literature reported that proportional evaluations offer superior comparability and stability, as they inherently possess an advantage in mitigating discrepancies among samples.^[^ ^22^, ^23^ ^]^ Therefore, there is an urgent need to develop new DNA technology that can achieve multi‐dimensional information on RNA concentration and its proportional variation in the RNA family to uncover tumor heterogeneity.

With the development of nucleic acid technology, DNA molecular devices have demonstrated compelling capabilities in biomolecular tracking and biological function deciphering due to their assembly controllability and operation programmability.^[^ ^24^, ^25^, ^26^ ^]^ To achieve discriminative ability at the single nucleotide level, various DNA molecular devices based on hybridization affinity regulation^[^ ^27^, ^28^ ^]^ or enzyme recognition pathway^[^ ^29^, ^30^ ^]^ have been developed. From the perspective of hybridization affinity regulation, a programmed strand displacement reaction process based on precise thermodynamic calculation has been reported to identify RNA mutations.^[^ ^31^ ^]^ Nevertheless, the thermodynamic calculation demands stationary temperature and ion content along with elaborate simulation and statistical analysis, making it unsuitable for popularization.^[^ ^32^, ^33^ ^]^ Owing to the remarkable insight of particular enzymes toward mismatch events, such as duplex‐specific nuclease (DSN), DNA within fully complementary RNA‐DNA heteroduplexes can be selectively cleaved, enabling a feasible approach to quantitatively identify RNA at single‐nucleotide resolution.^[^ ^34^ ^]^ It is conceivable that, through appropriate design, DSN may serve as a viable mediator for accurately screening a specific RNA from its highly homologous RNA family. Drawing inspiration from the aforementioned concept, we aim to construct a nucleic acid molecular device that intelligently combines hybridization affinity regulation and enzyme recognition to sequentially identify a highly homologous RNA family and a designated target RNA.

Herein, we design a detachable DNA assembly module that enables sequentially capturing a highly homologous RNA family and selectively screening for a target RNA, thus fulfilling a coinstantaneous investigation of the concentration and the proportion of the target RNA in its family population. This module is endowed with assembly and disassembly functions. In its assembly process, highly homologous RNA family members are extensively involved, which is beneficial for evaluating the total level of the RNA family. In contrast, its disassembly process only involves the target RNA, achieving the screen of the target RNA and the assessment of its expression abundance. Therefore, the proportion of the target RNA in its family population can be estimated by comparing the above information on concentration with the total family level. Additionally, the screening performance of the detachable DNA assembly module for the target RNA has been systematically validated via a DNA nanoflower, which can not only serve as an assessment tool to demonstrate the screening performance for target RNA, but also serve as a quantitative tool to further amplify the signal of the screened target RNA. By applying the highly homologous let‐7 family as the experimental paradigm, we discovered obvious differences in the proportion of let‐7a between normal MCF‐10A cells and tumor MCF‐7 cells. It is worth noting that this proportional information is frequently missed in bulk assays using conventional methods. Furthermore, utilizing machine learning algorithms to jointly analyze the obtained information on concentration and proportion, it has been substantiated that the combined analysis markedly enhances cellular discrimination. This represents a substantial advancement over the prevailing conventional techniques that rely solely on single‐concentration metrics. Hence, this work is expected to raise a novel perspective for a comprehensive understanding of tumor heterogeneity and to further guide personalized tumor therapy.

## 2 Results and Discussion

### 2.1 Operating Mechanism of the Detachable DNA Assembly Module

**Scheme 1:**
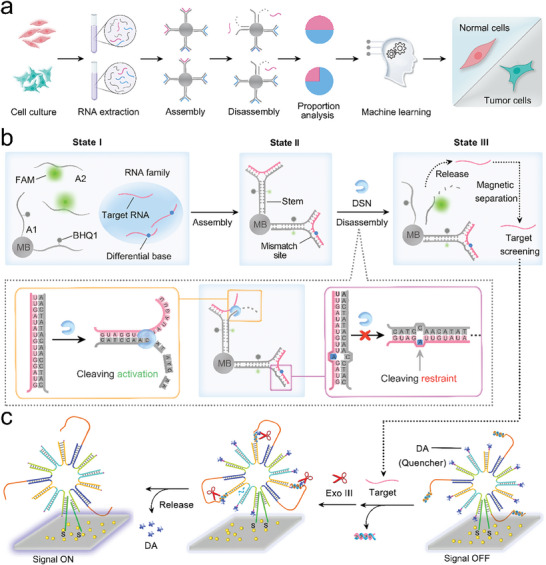
Detachable DNA assembly modules for RNA pinpoint screening. a) Process diagram for efficient cells discrimination based on the detachable DNA assembly module. b) Detachable DNA assembly module for selectively screening target RNA from its highly homologous RNA family through a sequential assembly and disassembly process. A1 modified BHQ1 is connected to magnetic beads, A2 modified with FAM, and the differential bases between other family members and target RNA are represented by blue dots. c) Using the DNA nanoflowers as amplifiers to evaluate the screening performance of the detachable DNA assembly module for target RNA. DA is modified as a quencher on DNA nanoflowers.

**Figure 1:**
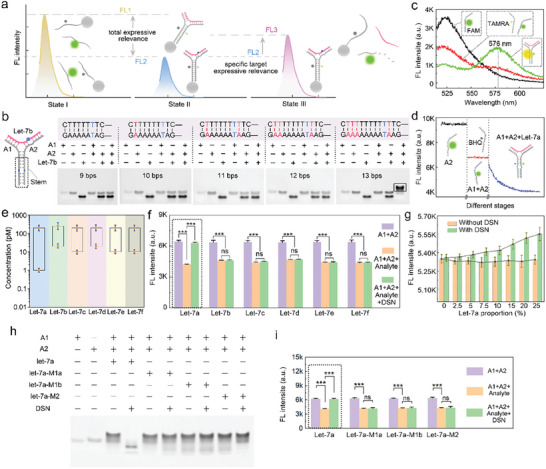
Study on the feasibility of detachable DNA assembly module for assembly and disassembly. a) The DNA module obtains the overall family level and concentration of target RNA through signal changes at different stages. b) Gel electrophoresis characterization of A1 and A2 with different stem lengths for completing module assembly. Red indicates extended sequence information and blue indicates mismatch site. c) The FRET system for feasibility verification of the module to assembly. The fluorescent receptor TAMRA was modified onto A1 instead of BHQ1, which constructed a FRET system with FAM. black line: A2; red line: A1 + A2; green line: A1 + A2 + let‐7a. d) Real‐time fluorescence analysis of the detachable DNA assembly module. e) Linear range of multiple family members for module assembly with components A1 and A2. f) Discrimination verification of the detachable DNA assembly module to the different analytes (i.e., let‐7a and other family members). The concentration of analyte in each experimental group was 20 nm. g) Discrimination of detachable DNA assembly module to let‐7a under different family proportions. In this experimental group, the total concentration of the let‐7 family was controlled at 10 nm, and the concentration of let‐7a was adjusted to different proportions while keeping the concentration of each other family members consistent. ***p < 0.001; ns, non‐significant in the *t*‐test. h) Gel electrophoresis characterization and i) fluorescence analysis of the detachable DNA assembly module to let‐7a and its analogs.

As shown in **Scheme** **1**a, this work aims to use the detachable DNA assembly module to obtain the concentration and proportion of a specific RNA in its highly homologous RNA family through sequential assembly and disassembly processes, thereby achieving efficient discrimination of cells by utilizing machine learning algorithms. The operating mechanism of this assembly module is shown in Scheme 1b: The black hole quencher 1 (BHQ1)‐labeled A1 immobilized on magnetic beads (MB), and the 6‐carboxyfluorescein (FAM)‐labeled A2 as the DNA assembly module components were designed to distinguish the highly homologous RNA family and target RNA. First, the freely diffused components A1 and A2 emitted strong fluorescence in the initial state (State I). When the highly homologous RNA family existed, the components A1 and A2 exhibited robust hybridization affinity with the RNA family and rapidly completed module assembly, resulting in dramatic fluorescence quenching (State II). Therefore, the total family level can be tracked by the fluorescence quenching from state I to state II. With the intervention of DSN, the portion that completely hybridized with target RNA in A2 was degraded, triggering the disassembly of these modules and the release of target RNA, so the fluorescence signal was partially restored (State III). The fluorescence recovery from state II to state III refers to the abundance of target RNA. Novelly, the proportion of target RNA in its family population could be tracked via the signal variations in module state transitions (**Figure** **1**a). Otherwise, due to the high sensitivity of DSN to mismatch events, other family members of target RNA were preserved on their modules which were linked on MB. As such, an efficient screen of target RNA from its homologous family members could be realized by applying a magnetic field. Additionally, we customized DNA nanoflowers as the signal amplification tool to evaluate the screening ability of the assembly module for target RNA (Scheme 1c). The walking strand on the DNA nanoflower was activated by the target RNA and continuously walked along the track strand with the assistance of Exonuclease III (Exo III).

### 2.2 Continuous State Transition of the Detachable DNA Assembly Module

The let‐7 family with highly homologous and specifically let‐7a was chosen as the validation model to confirm the continuous assembly and disassembly performance of the detachable DNA assembly module, which was verified through the polyacrylamide gel electrophoresis (PAGE) and fluorescence characterization. To achieve extensive capture of the let‐7 family population by the assembly module, we first selected let‐7b, which differs the most from let‐7a among the highly expressed family members (as discussed in the Supporting Information), as the optimization model to investigate the reasonable structural configuration of the module for assembly. As shown in Figure 1b, various stem lengths ranging from 9 bps to 13 bps were designed and validated separately. It should be noted that a mismatched site was set on the stem domain, which prevented DSN from disassembling the assembly module through this domain. It was observed that the 13 bps stem length rendered the band of the DNA module (marked in black box), suggesting that this stem length was appropriate for forming the assembly module. To verify the assembly feasibility of the module, we constructed a fluorescence‐resonance energy transfer (FRET) system. As shown in Figure 1c, target let‐7a triggered the FRET between the fluorescence donor (FAM) and the fluorescence receptor molecule (tetramethylrhodamine, TAMRA) labeled on the complementary stem, indicating the successful formation of these modules. This was further confirmed through the continuous quenching effect triggered by let‐7a in fluorescence kinetics analysis (Figure 1d). These results proved the feasibility of individual let‐7a triggering module assembly. Next, we extended the scope to the let‐7 family. As shown in Figure 1e, multiple family members (i.e., let‐7a ∼ let‐7f) demonstrated their respective ability to participate in module assembly across a wide range of concentrations. Each linear relationship was listed in Figure S1 (Supporting Information), which suggested that the assembly modules are capable of broadly capturing the let‐7 family population during their assembly process.

We next validated the disassembly property of the assembly module in response to DSN. As depicted in Figure 1f, all these let‐7 family members formed modules with A1 (labeled with fluorescence quencher BHQ1) and A2 (labeled with fluorescence donor FAM). However, compared to the modules assembled by other family members, only the let‐7a‐involved module exhibited a fluorescence recovery upon DSN treatment, implying the disassembly of the module. Even in the mixture of the let‐7 family, the assembly module still demonstrated satisfactory discriminability toward let‐7a (Figure S2, Supporting Information). Moreover, the sensitivity of the assembly module to let‐7a with different family proportions was also investigated. As shown in Figure 1g, we adjusted the let‐7a to different proportions while keeping the total concentration of the let‐7 family constant. Before DSN treatment, the fluorescence signals in each experimental group were similar, implying that other family members have a similar module assembly ability to let‐7a. However, upon DSN treatment, the fluorescence increased with the let‐7a proportion. These results provided preliminary evidence for the ability of the detachable DNA assembly module to identify highly homologous let‐7 family and to screen let‐7a. By randomly mutating one (let‐7a‐M1a and let‐7a‐M1b) and two bases (let‐7a‐M2) of the target let‐7a, we obtained several let‐7a analogs and further validated the recognition performance of the detachable DNA assembly module on them. First, gel electrophoresis analysis showed that these analogs could all participate in module assembly (Figure 1h). However, with the addition of DSN, only these let‐7a‐assembled modules were disassembled, while the other modules remained, indicating the high specificity of these modules to let‐7a. In the further fluorescence analysis, all let‐7a and its analogs (let‐7a‐M1a, let‐7a‐M1b, and let‐7a‐M2) triggered fluorescence quenching, while only these let‐7a‐assembled modules achieved signal recovery (Figure 1i). These results fully demonstrated that the detachable DNA assembly module has a single‐base level detection limit for recognizing let‐7a within the let‐7 family.

We next analyzed the feasibility of applying this assembly module in other highly homologous RNA families. By choosing the miRNA‐34 family and miRNA‐181 family as target RNA families, and the miRNA‐34a and the miRNA‐181a as specific target RNA, we constructed two corresponding detachable DNA assembly modules (Figures S3 and S4, Supporting Information). Through gel electrophoresis and fluorescence analysis, the two proposed modules could respectively respond to miRNA‐34a and miRNA‐181a for assembly and disassembly, while other family members cannot participate in the module disassembly process, demonstrating the versatility of the detachable DNA assembly module for analyzing multiple RNA families. Furthermore, we used the classic single base repeat site NR‐21 as a model validated the possibility of the proposed module in microsatellite instability analysis (Figure S5, Supporting Information), demonstrating the potential of detachable DNA assembly module in identifying events such as insertion and deletion of microsatellite sequences.

### 2.3 Proposal and Validation of Evaluation Tools

**Figure 2:**
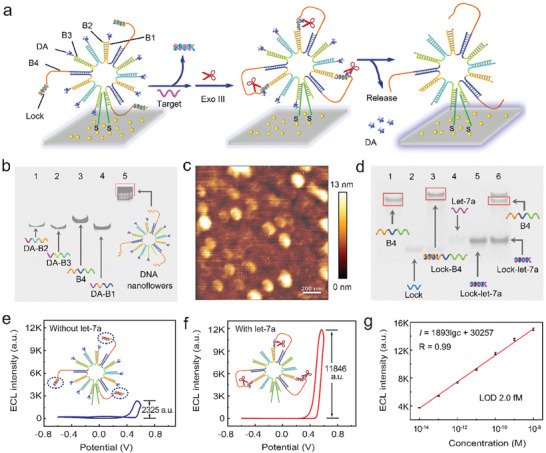
Feasibility of DNA nanoflowers for let‐7a quantitative analysis. a) Reaction mechanism of the ordered DNA nanoflowers. b) PAGE analysis of the self‐assembly of the DNA nanoflowers. lane 1, DA‐B2; lane 2, DA‐B3; lane 3, B4; lane 4, DA‐B1; lane 5, the DNA nanoflowers. c) AFM images of ordered DNA nanoflower. d) PAGE analyses of the lock‐B4 for let‐7a recognition. lane 1, B4; lane 2, lock; lane 3, target let‐7a; lane 4, the mixture of lock and B4; lane 5, the mixture of target let‐7a and lock; lane 6, the mixture of B4, lock, and target let‐7a. e,f) Responses of the DNA nanoflowers‐based biosensor (e) without and (f) with incubation with 100 pm let‐7a. g) Linear fitting of let‐7a quantification using the DNA nanoflower‐based biosensor. The concentration of let‐7a increased from 10 fm to 10 nm.

To comprehensively evaluate the screening performance of the assembly module for let‐7a in complex samples, the DNA nanoflowers were customized as signal amplifiers to quantitatively analyze the screened target RNA (**Figure** **2**a). The detailed mechanism is shown in Figure S6 (Supporting Information). The DNA nanoflowers were self‐assembled with single‐stranded DNAs (B1, B2, B3, and B4) through base pairing. Dopamine (DA) was modified on B1, B2, and B3 to quench the initial signal, while B4 was blocked with a DNA lock. When the target let‐7a reacted with the lock through a toehold‐mediated strand displacement reaction, B4 was then relieved, which subsequently reacted with adjacent B1, B2, or B3 to trigger a successive Exo III‐mediated enzymatic cleavage reaction. As such, DA was removed and the signal was restored (Figure 2a). Next, we validated the successful assembly of DNA nanoflowers. The formation of DNA nanoflowers and the response of lock‐linked B4 (lock‐B4) to let‐7a were verified by PAGE analysis. As shown in Figure 2b, a band in lane 5 with obviously slower mobility than that of the component DNAs (lines 1–4) was observed, indicating the formation of DNA nanoflowers. The atomic force microscope (AFM) image showed the monodispersed DNA nanoflowers with an average diameter of ≈100 nm and a height of ≈13 nm (Figure 2c).

Next, we validated the responsiveness of lock‐B4 toward let‐7a. As shown in Figure 2d, when lock‐B4 reacted with let‐7a, clear single‐stranded B4 and double‐stranded lock‐let‐7a were observed in lane 6, indicating that let‐7a can activate DNA nanoflowers for signal amplification. After employing electrochemiluminescence (ECL) and cyclic voltammetry characterization to supervise the assembly process of DNA nanoflowers into the biosensor (Figure S7a,b, Supporting Information), we tested the response of the DNA nanoflower‐based biosensor toward let‐7a. The DA‐labeled DNA nanoflowers exhibited a low signal due to the quenching of DA (Figure 2e), while after treatment with let‐7a, a significant signal enhancement was observed (Figure 2f). This indicated that the DNA nanoflowers could be activated by let‐7a with their signal turned “on”. Furthermore, the signal linearly increased with the concentration of let‐7a and the calculated limit of detection (LOD) was 2 fm (Figure 2g), which was two orders of magnitude lower than that of the detachable DNA assembly module (Figure S1a, Supporting Information, LOD of 0.62 nm). Collectively, these results validated the feasibility of the DNA nanoflowers to evaluate the screening performance of the detachable assembly module for let‐7a.

### 2.4 Performance Evaluation of the Assembly Module for let‐7a Screening in Complex Samples

**Figure 3:**
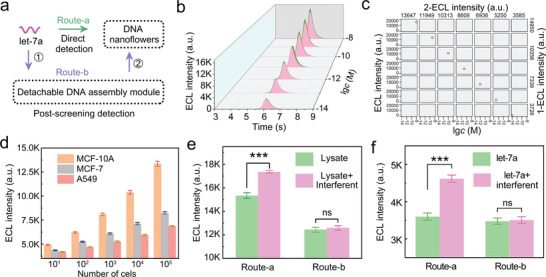
Performance evaluation of the assembly module for let‐7a screening in complex samples. a) Diagram of evaluating the screening performance of the DNA nanoflowers to the detachable DNA assembly modules via two routes. b) ECL characterization of route‐a (in green) and route‐b (in pink) to different concentrations of let‐7a (10 fm to 10 nm). c) Correlation coefficient scatter plot of linear response fitting of route‐a and route‐b. d) Response intensity of route‐b to let‐7a in the lysate of MCF‐10A cells, MCF‐7 cells, and A549 cells. e) Specific response of let‐7a in MCF‐10A lysate through the route‐a and the route‐b. The number of cells is 10^4^. f) Specific validation of route‐a and route‐b for let‐7a analysis. The concentration of let‐7a was 1 pm, and other family members were used as interferences in this experimental group, with a total concentration of 10 pm. ***p < 0.001; ns, non‐significant in *t*‐test.

The screening performance of the detachable DNA assembly module for let‐7a was evaluated by combining the assembly module with the DNA nanoflowers. As shown in **Figure** **3**a, the let‐7a standard solution was quantified through two different routes (route‐a and route‐b). Unlike the direct quantification of let‐7a by DNA nanoflowers (route‐a), we conducted a post‐screening process by the detachable DNA assembly module before quantifying let‐7a by the DNA nanoflowers (route‐b). Despite undergoing an additional screening process, route‐b still responded well to different concentrations of let‐7a solution (Figure 3b; Figure S8a, Supporting Information, in pink), which was similar to the result of route‐a (Figure S8b, Supporting Information). Meanwhile, through the linear scatter fitting analysis of the above results, a Pearson correlation of 0.996 was obtained (Figure 3c), which indicated that the assembly module enables efficient capture and separate let‐7a.

We next applied route‐b to let‐7a analysis in cell lysate. As shown in Figure 3d, route‐b outputted different fluorescence signals for the lysates with different cell quantities, which was consistent with previous reports.^[^ ^19^, ^35^ ^]^ In addition, we compared the results of the lysate analysis between route‐a and route‐b. As shown in Figure 3e, when only detecting the lysate (in green), the signal of the route‐a was significantly higher than that of the route‐b. Furthermore, when other family members as interferent were added into the lysate (in pink), the signal enhancement was also observed in route‐a, and there was no noticeable signal change in route‐b. This may be attributed to the excellent discrimination of the detachable DNA assembly module to let‐7a, effectively eliminating other interferents. To confirm the above hypothesis, we conducted a control experiment by adding other family members as interferent to the let‐7a standard solution (Figure 3f). As expected, with the addition of other family members, the signal in route‐a continued to increase, while there was no significant difference in the signal of route‐b. The above results proved that the detachable DNA assembly module can efficiently screen let‐7a in complex samples.

### 2.5 Intracellular Imaging Applications of Detachable DNA Assembly Module

**Figure 4:**
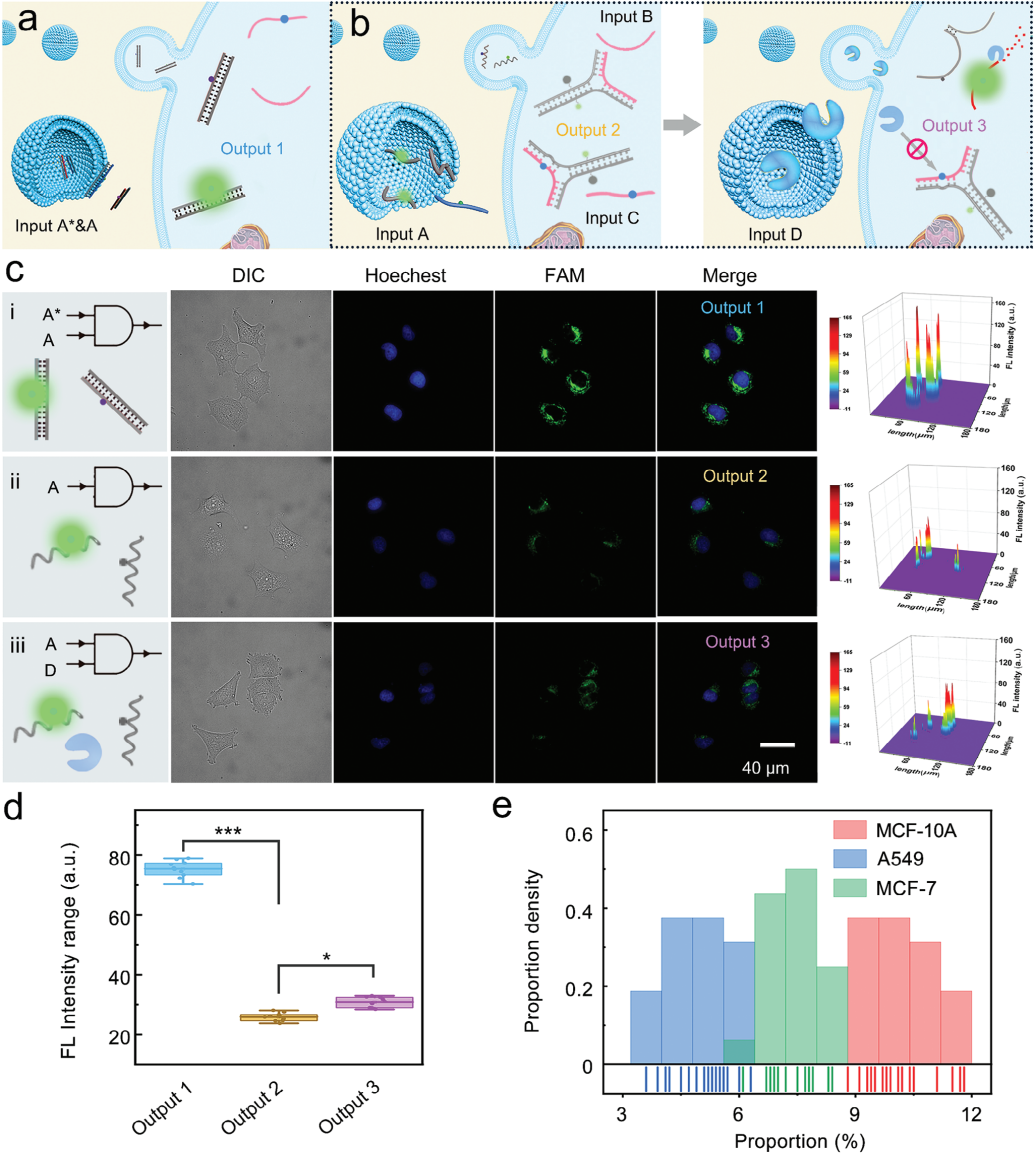
Feasibility of the detachable DNA assembly module in cells. a,b) Schematic diagram of the logic gated screening route for a) let‐7 family capture and b) let‐7a release. c) Fluorescence images of MCF‐7 incubated with i) input A and input A*, ii) input A alone, and iii) input A and input D. d) Gray value quantization statistics of the fluorescence output range of MCF‐7 cells treated in Figure 4c. *n* = 4, *p < 0.05, ***p < 0.001 in *t*‐test. e) Proportion range and frequency of let‐7a detected in MCF‐10A cells, MCF‐7 cells, and A549 cells.

To explore the feasibility of the detachable DNA assembly module to discriminate let‐7a and its family members in living cells, a logic gate screening route was proposed. As shown in **Figure** **4**a,b, the module components (i.e., A1 and A2) were defined as input A. The intracellular let‐7a and other family members were defined as input B and input C, respectively. DSN was defined as input D. To obtain the total level of the let‐7 family, the silence DNA sequence corresponding to the module components was introduced as input A*, and the logical path diagram was organized in Figure S9 (Supporting Information). As shown in Figure 4c, when input A was blocked by input A* (input A&A*), a bright fluorescence emission in MCF‐7 cells was observed (i.e., output 1) because A1 and A2 were in a free state, unable to assemble into a module. The individual input A led to weak output fluorescence (i.e., output 2), while input A and input D incubation resulted in a fluorescence enhancement (i.e., output 3). Compared to output 2, output 3 had been improved to a certain extent (Figure 4d; Figure S10, Supporting Information), indicating that the assembly module can screen let‐7a in cells.

To confirm that the improvement in output 3 indeed originated from the precise screen of let‐7a, silencing, and interference experiments were carried out, respectively. Silencing let‐7a resulted in no statistically significant signal alteration upon DSN treatment, even when similar molecules were introduced (Figure S11, Supporting Information), which elucidated the unique discriminatory capability of the assembly module toward let‐7a. On this basis, the application feasibility of the assembly module in A549 cells and MCF‐10A cells was also studied (Figures S12 and S13, Supporting Information). As expected, the detachable DNA assembly module responded sensitively to the intracellular let‐7a in the above two cell lines. After statistical analysis of the above proportional information, we found significant differences in the proportion of let‐7a among MCF‐10A cells, MCF‐7 cells, and A549 cells, respectively (Figure 4e). It can be observed that even between normal mammary epithelial cells and breast cancer cells, the difference in the proportion of let‐7a is still significant. These proportional differences are often missed in bulk analysis of conventional methods. Hence, the above findings demonstrated the application potential of the detachable DNA assembly module in cytosolic RNA surveillance. In addition, to verify the universality of the detachable DNA assembly module in analyzing different cell types, we applied it to analyze the expression of let‐7a in human pancreatic duct epithelial cells (hTERT‐HPNE) and pancreatic cancer cells (PANC‐1) (Figure S14 and S15, Supporting Information). The results showed a significant difference in the proportion of let‐7a between hTERT‐HPNE and pancreatic cancer cells, confirming the universality of the detachable DNA assembly module in cell analysis. Furthermore, we applied the detachable DNA assembly module to the dynamic monitoring of A549 cells under the treatment of chemotherapy drug cisplatin (Figure S16, Supporting Information), and found the continuous increase in the proportion of let‐7a in A549 cells within 36 h of cisplatin treatment, demonstrating the superiority of the assembly module in dynamic monitoring of cellular RNA alterations.

### 2.6 Detachable DNA Assembly Module for Cells Discrimination

**Figure 5:**
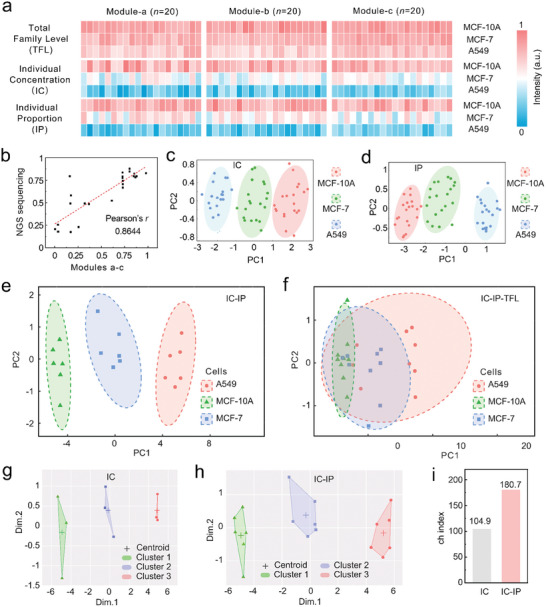
Combinatory analysis of the concentration and the proportion information obtained from the assembly module via machine learning algorithms for cells discrimination. Normalized heat maps obtained from a) three assembly modules (i.e., Module‐a, Module‐b, and Module‐c) regarding the TFL, IC, and IP in cell lysate analysis. b) Correlation of these three assembly modules with NGS analysis for TLF, IC, and IP evaluation of three cell lines. c,d) PCA score plot of c) IC and d) IP from the detachable DNA assembly module for cells discrimination. e,f) PCA score plot of e) IC‐IP and f) IC‐IP‐TFL for cells discrimination. g,h) K‐means clustering classification graph based on the PCA results at the g) IC level and h) IC‐IP level. The centroid corresponding to each cluster was marked with a plus sign in the corresponding color. i) The ch index of the K‐means clustering classification graph at the IP level and IC‐IP level.

To reveal the application potential of the DNA assembly module in cellular discrimination, we designed three assembly modules (i.e., Module‐a, Module‐b, and Module‐c) that specifically target miRNAs (i.e., let‐7a, let‐7b, and let‐7c) for discriminating between three cell lines (i.e., MCF‐10A, MCF‐7, and A549 cells). Before conducting cells discrimination, we verified the analytical ability of Module‐b and Module‐c for let‐7b and let‐7c, respectively (Figure S17a,b, Supporting Information). Then, the total family level (TFL), individual concentration (IC), and individual proportion (IP) data of these three cell lines were obtained by the three modules and normalized separately (**Figure** **5**a). We performed next‐generation the sequencing (NGS) analysis on these types of cells (Figure S18a, Supporting Information). As shown in Figure 5b, three assembly modules‐based data (Modules a–c) exhibited a strong correlation with the sequencing data (Pearson's *r* = 0.8644), demonstrating the reliability of the detachable DNA assembly modules in analyzing family information and screening target RNA. Furthermore, we performed principal component analysis (PCA) for cell discrimination across three cell lines. As shown in Figure 5c,d, three cell lines has been effectively distinguished based on the IC and IP obtained by detachable DNA assembly modules. The NGS analysis‐derived IC (IC‐seq) and IP (IP‐seq) also exhibited dispersion views similar to those of IC and IP results (Figure S18b,c, Supporting Information). Therefore, these results fully demonstrated the superior performance of the detachable DNA assembly modules in analyzing RNA family information and screening target RNA. Building on this, we attempted to perform a combined analysis of the information obtained from the detachable DNA assembly modules. As shown in Figure 5e, the combination of IC and IP (IC‐IP) as indicators achieved effective discrimination among the three cell lines. However, the cells discrimination failed using the combination of IC, IP, and TFL (IC‐IP‐TFL, Figure 5f). The result implied that IC and IP were highly informative for cells discrimination, while the TFL analysis masked these cellular heterogeneities.

To study the role of IC and IP in combined analysis, we further studied the cells discrimination effect of the PCA results above using the k‐means clustering algorithm. The IC and IP from three modules were separately evaluated by PCA (Figure S19, Supporting Information) to provide the k‐means clustering view. As shown in Figure 5g and Figure S20a (Supporting Information), both independent IC and IP supported discrimination among the three cell lines. The Calinski‐Harabaz (ch) index based on the k‐means clustering view of IC and IP was similar (104.9 and 97.3, Figure 5h; Figure S20b, Supporting Information), indicating that IP also has independent significance for cells discrimination. However, when we executed k‐means clustering of IC‐IP (Figure 5h), a more centralized clustering view was obtained, and the ch index was improved to 180 (Figure 5i). The increase in the ch index demonstrated convincingly that the conjoint analysis of IC and IP holds clear advantages in cells discrimination over IC alone. These results validated the superiority of the detachable DNA assembly module in cellular discrimination, stemming from their ability to uncover more heterogeneous information than conventional methods under the same sample size.

## 3 Conclusion

We designed a detachable DNA assembly module that not only enables precise screening of a specific RNA from a highly homologous RNA family but also allows for the assessment of the proportion of the RNA within its family population, thus achieving efficient cells discrimination. Compared to conventional methods that are based only on the concentration, we used the detachable DNA assembly module to obtain proportional information, therefore remarkably improving the cellular discrimination performance. Simultaneously, we found a significant proportional difference in let‐7a between normal MCF‐10A cells and tumor MCF‐7 cells. Through the analysis of differential RNA expression, differences in RNA expression proportions between different tumor samples can be elucidated, leading to a better understanding of tumor heterogeneity. This will contribute to the identification of distinct tumor subtypes, clinical subgroups, and potential prognostic biomarkers.

## 4 Experimental Section

### Pretreatment of Freeze‐Dried DSN Enzyme

First, DSN storage buffer was added to the lyophilized DSN enzyme (5 µL per 10 units), and the tube wall was flicked evenly and separated instantly to avoid foam. Then, the same volume of 100% glycerol was added to the tube, gently bounced the wall evenly, and separated instantly. Finally, the DSN solution was saved at −20 °C.

### Construction of the Biosensor

Before the modification of the glassy carbon electrode (GCE, *Φ* = 4 mm), GCE was pretreated with 0.3 and 0.5 µm alumina polishing powder successively. Then, ultrasonic treatment toward GCE was carried out using ethanol and ultrapure water for 5 min to remove the residual alumina powder. The gold nanoparticles (Au NPs, 10 µL) solution was dripped on the treated GCE surface and dried naturally. Then, the 10 µL ordered DNA nanoflower (1 µm) was incubated on the surface of GCE overnight at 4 °C. After washing the physically adsorbed ordered DNA nanoflower with ultrapure water, 10 µL HT (1 m) was incubated on the surface of the resultant GCE for 30 min. Finally, the modified biosensor was gently washed and placed at 4 °C for further use.

### Fluorescence Detection Process

First, DNA and RNA were dissolved and diluted to different concentrations by Tris‐EDTA (TE, pH 8.0) buffer and hybridization buffer (HB, pH 7.4), respectively, and the resultant solution was heated to 95 °C and slowly cooled to 25 °C. Subsequently, 45 µL carboxyl‐modified A1 was treated with 5 µL mixture of N‐(3‐dimethylaminopropyl)‐N′‐ethylcarbodiimide hydrochloride (EDC) and N‐hydroxy succinimide (NHS) (EDC: NHS was 4:1), which was then reacted with amino‐modified Fe_3_O_4_ magnetic microsphere (MB) at 4 °C overnight to modify A1 onto the surface of MB. Furthermore, 2 µL resultant A1‐MB (A1, 4 µm), 2 µL A2 (4 µm), and 2 µL target in different concentrations were mixed and diluted to 20 µL by HB for 2 h at 37 °C. Similarly, when DSN was needed in a parallel experimental group, 2 µL target at different concentrations, 2 µL A1‐MB (A1, 4 µm) and 2 µL A2 (4 µm) was mixed for 2 h. Then, 1 µL 10 × DSN master buffer and 1 µL DSN solution were added to the above solution for 1 h to shear the detachable DNA assembly module. After that, 4 µL 2 × DSN stop buffer was added to the resultant solution for 5 min. Finally, the mixture was diluted to 20 µL by HB. The A1 and A2 were modified with black hole quencher 1 (BHQ1) and carboxyfluorescein (FAM), respectively. The fluorescence intensity was acquired by the FL‐7000 fluorescence spectrophotometer, and the voltage and the slit width were set as 950 V and 5.0 nm, respectively. After fluorescence detection, the reaction solution was magnetically separated and the supernatant was collected for further quantification by ordered DNA nanoflower.

### Statistical Analysis

All data were expressed as the mean ± standard deviation (SD) and 95% confidence intervals. Unless otherwise stated, all data were obtained by triplicate measurements. Pre‐processing and statistical analysis relevant information was indicated in the experimental section or in the figure legends. Normalize and statistically analyze the mean extreme differences of three types of cells under different parameters (TFL, IC, IP). Two‐tailed t‐test were used to compare the statistical significance between two groups. The resulting p‐values have been described in the corresponding figures. The linear scatter fitting analysis was performed by SPSS 14.0. Conducted sequencing data analysis using Trimmatic‐0.38 and miRDeep2. Statistical analyses and data visualization of machine learning were performed by R package.

## Conflict of Interest

The authors declare no conflict of interest.

## Supporting information



Supporting Information

## Data Availability

The data that support the findings of this study are available from the corresponding author upon reasonable request.

## References

[1] S. L. Topalian , F. S. Hodi , J. R. Brahmer , C. T. Harbison , J. F. Grosso , M. Sznol , JAMA oncology 2019, 5, 1411. 31343665 10.1001/jamaoncol.2019.2187 PMC6659167

[2] J. L. Vos , J. B. W. Elbers , O. Krijgsman , X. H. Qiao , Y. Lubeck , I. M. Seignette , L. A. Smit , S. M. Willems , Nat. Commun. 2021, 12, 1. 34937871 10.1038/s41467-021-26472-9 PMC8695578

[3] Z. H. Wu , D. Q. Huang , J. L. Wang , Y. J. Zhao , W. J. Sun , X. Shen , Adv. Sci. 2024, 11, 2304160. 10.1002/advs.202304160 PMC10767453 37946674

[4] L. Keller , K. Pantel , Nat. Rev. Cancer 2019, 19, 553. 31455893 10.1038/s41568-019-0180-2

[5] G. J. Goodall , V. O. Wickramasinghe , Nat. Rev. Cancer 2021, 2, 22. 10.1038/s41568-020-00306-0 33082563

[6] H. N. Gruner , M. T. McManus , Nat. Rev. Genet. 2021, 22, 448. 33824487 10.1038/s41576-021-00346-8

[7] S. Zhao , S. H. Zhang , H. J. Hu , Y. C. Cheng , K. X. Zou , J. Song , J. Q. Deng , L. L. Li , X. B. Zhang , G. L. Ke , J. S. Sun , Angew. Chem., Int. Ed. 2023, 62, e202303121. 10.1002/anie.202303121 37078239

[8] H. R. Chen , M. L. Su , Y. M. Lei , Z. X. Ye , Z. P. Chen , P. Y. Ma , R. Yuan , Y. Zhuo , C. Y. Yang , W. B. Liang , J. Am. Chem. Soc. 2023, 23, 12812. 10.1021/jacs.3c03626 37249527

[9] D. Zhao , K. R. Wu , S. Sharma , F. Xing , A. Tyagi , R. Deshpande , M. Wabitsch , Y. Y. Mo , K. Watabe , Nat. Commun. 2022, 13, 1. 36517516 10.1038/s41467-022-35305-2 PMC9751138

[10] J. Tang , X. Wang , D. S. Xiao , S. Liu , Y. G. Tao , Mol. Cancer 2023, 22, 1. 36750826 10.1186/s12943-023-01724-y PMC9903551

[11] T. M. Raimondo , K. Reed , D. N. Shi , R. Langer , Cell 2023, 186, 1535. 37059063 10.1016/j.cell.2023.02.031

[12] D. W. Kim , K. J. Tu , A. Wei , A. J. Lau , A. Gonzalez‐Gil , T. Y. Cao , K. Braunstein , J. P. Ling , P. C. Wong , J. C. Troncoso , S. Blackshaw , R. L. Schnaar , T. Li , Mol. Neurodegener. 2022, 17, 1. 36536457 10.1186/s13024-022-00589-x PMC9762062

[13] Y. T. Zou , F. Ye , Y. A. Kong , X. Q. Hu , X. P. Deng , J. D. Xie , C. L. Song , X. Q. Ou , W. W. Tian , Y. H. Tang , C. W. Wong , Z. S. Chen , X. H. Xie , H. L. Tang , Adv. Sci. 2023, 10, 2203699. 10.1002/advs.202203699 PMC9929130 36529697

[14] Z. Q. Zhao , D. Zhang , F. Q. Yang , M. R. Xu , S. L. Zhao , T. T. Pan , Q. F. Wu , Q. Tu , P. Zhou , R. Li , J. Kang , L. Zhu , F. Gao , Y. Q. Wang , Z. H. Xu , Cell Res. 2022, 32, 425. 35273378 10.1038/s41422-022-00635-9 PMC9061815

[15] F. Xiao , X. F. Fang , H. Y. Li , H. B. Xue , Z. X. Wei , W. K. Zhang , Y. L. Zhu , L. Lin , Y. Zhao , C. F. Wu , L. L. Tian , Angew. Chem., Int. Ed. 2022, 61, e202115812. 10.1002/anie.202115812 35064628

[16] S. Kang , H. Ahn , C. Park , W. H. Yun , J. G. Jeong , Y. J. Lee , D. W. Kim , Adv. Sci. 2023, 10, 2300462. 10.1002/advs.202300462 PMC10238211 37066794

[17] Z. H. Qing , J. Y. Xu , J. L. Hu , J. Zheng , L. He , Z. Zou , S. Yang , W. H. Tan , R. H. Yang , Agnew. Chem. 2019, 131, 11698.

[18] T. W. J. Zhou , H. Li , K. K. Zhang , F. L. Wang , X. Chu , J. H. Jiang , J. Am. Chem. Soc. 2021, 143, 14394. 34431301 10.1021/jacs.1c07719

[19] T. Hao , W. X. Li , G. H. Wang , T. Ye , Y. Jie , S. T. Zhou , X. Y. Yu , B. Li , Y. L. Dai , Adv. Sci. 2023, 10, 2301661.

[20] T. F. Wang , E. Calvo‐Roitberg , J. M. Rembetsy‐Brown , M. G. Fang , J. Sousa , Z. J. Kartje , P. M. Krishnamurthy , J. Lee , M. R. Green , A. A. Pai , J. K. Watts , Nucleic Acids Res. 2022, 50, 12657. 36511872 10.1093/nar/gkac1108 PMC9825156

[21] F. Y. Ding , S. Cocco , S. Raj , M. Manosas , T. T. T. Nguyen , M. M. Spiering , D. Bensimon , J. F. Allemand , V. Croquette , Nucleic Acids Res. 2022, 50, 12082. 36478056 10.1093/nar/gkac1113 PMC9757040

[22] L. A. B. Wilson , S. R. K. Zajitschek , M. Lagisz , J. Mason , H. Haselimashhadi , S. Nakagawa , Nat. Commun. 2022, 13, 7502. 36509767 10.1038/s41467-022-35266-6 PMC9744842

[23] J. B. Smaers , R. S. Rothman , D. R. Hudson , D. K. N. Dechmann , D. de Vries , J. C. Dunn , J. G. Fleagle , K. Safi , Sci. Adv. 2021, 7, eabe2101. 33910907 10.1126/sciadv.abe2101 PMC8081360

[24] M. Stasi , A. Monferrer , L. Babl , S. Wunnava , C. F. Dirscherl , D. Braun , P. Schwille , H. Dietz , J. Boekhoven , J. Am. Chem. Soc. 2022, 144, 21939. 36442850 10.1021/jacs.2c08463 PMC9732876

[25] J. L. Liu , C. Zhang , J. X. Song , Q. Zhang , R. J. Zhang , M. Z. Zhang , D. Han , W. H. Tan , Adv. Sci. 2023, 10, 2206343. 10.1002/advs.202206343 PMC10369254 37116171

[26] J. L. Wang , J. Li , Y. Chen , R. T. Liu , Y. X. Wu , J. B. Liu , X. H. Yang , K. M. Wang , J. Huang , Nano Lett. 2022, 22, 8216. 36194690 10.1021/acs.nanolett.2c02934

[27] A. Poddar , M. S. Azam , T. Kayikcioglu , M. Bobrovskyy , J. C. Zhang , X. Q. Ma , P. Labhsetwar , J. Y. Fei , D. Singh , Z. Luthey‐Schulten , C. K. Vanderpool , T. Ha , Nat. Commun. 2021, 12, 1. 33558533 10.1038/s41467-021-21144-0 PMC7870926

[28] G. He , J. Li , H. N. Ci , C. M. Qi , X. F. Guo , Angew. Chem. 2016, 128, 9182.

[29] Y. B. Wang , W. T. Cottle , H. B. Wang , M. Gavrilov , R. S. Zou , M. T. Pham , S. Yegnasubramanian , S. Bailey , T. Ha , Nat. Commun. 2022, 13, 7776. 36522352 10.1038/s41467-022-35476-y PMC9755149

[30] W. Zhang , Y. Q. Mu , K. J. Dong , L. Zhang , B. Yan , H. Hu , Y. W. Liao , R. Zhao , W. Shu , Z. X. Ye , Q. Q. Sun , L. J. Li , H. B. Wang , X. J. Xiao , Nucleic Acids Res. 2022, 50, 12674. 36484104 10.1093/nar/gkac1144 PMC9825152

[31] T. Zhang , R. J. Deng , Y. X. Wang , C. Y. Wu , K. X. Zhang , C. Y. Wang , N. Q. Gong , R. Ledesma‐Amaro , X. C. Teng , C. R. Yang , T. Xue , Y. Zhang , Y. Hu , Q. He , W. M. Li , J. H. Li , Nat. Biomed. Eng. 2022, 6, 957. 35835993 10.1038/s41551-022-00907-0

[32] C. Y. Zhou , D. L. Yang , S. Sensale , P. Sharma , D. F. Wang , L. Yu , G. Arya , Y. G. Ke , P. F. Wang , Sci. Adv. 2022, 8, eade3003. 36399380 10.1126/sciadv.ade3003 PMC9674029

[33] M. R. Adendorff , G. Q. Tang , D. P. Millar , M. Bathe , W. P. Bricker , Nucleic Acids Res. 2022, 50, 717. 34935970 10.1093/nar/gkab1246 PMC8789063

[34] T. W. Nam , Y. Park , Y. S. Jung , H. G. Park , ACS Nano 2022, 16, 11115. 35704843 10.1021/acsnano.2c03806

[35] J. Li , S. Y. Liu , J. L. Wang , R. T. Liu , X. H. Yang , K. M. Wang , J. Huang , Nucleic Acids Res. 2022, 50, e40. 34935962 10.1093/nar/gkab1258 PMC9023253

