# Supplementary material for: Detachable DNA Assembly Module to Dissect Tumor Cells Heterogeneity via RNA Pinpoint Screening
Source: Adv Sci (Weinh). 2024 Oct 18;11(46):2401253. doi: 10.1002/advs.202401253 (PMC11633503; doi:10.1002/advs.202401253)
Supplement: Supplementary file 1 — Supporting Information [file ADVS-11-2401253-s001.docx]

**Detachable DNA Assembly Module to Dissect Tumor Cells Heterogeneity via RNA Pinpoint Screening**

Wei Liu^[a]^, Ni Liao^[b]^, Yanmei Lei^[a,c]^, Wenbin Liang^[a]^, Xia Yang^[a]^, Ruo Yuan^[a]^, Chaoyong Yang*^[c,d]^, and Ying Zhuo*^[a]^

*^[a]^ Key Laboratory of Luminescence Analysis and Molecular Sensing (Southwest University), Ministry of Education, College of Chemistry and Chemical Engineering, Southwest University, Chongqing 400715, PR China*

*^[b]^ College of Biological and Chemical Engineering, Panzhihua University Panzhihua, 617000, PR China*

*^[c]^ Institute of Molecular Medicine, Renji Hospital, School of Medicine, Shanghai Jiao Tong University, Shanghai 200127, PR China*

*^[d]^ The MOE Key Laboratory of Spectrochemical Analysis and Instrumentation, Department of Chemical Biology, College of Chemistry and Chemical Engineering, Xiamen University, Xiamen 361005, PR China*

**Supplemental Information**

**List of contents:**

**Supplemental discussions**

| Reagents | S1 |
| --- | --- |
| Apparatus | S2 |
| Cells Culture and Lysate Extract | S2 |
| Electrochemiluminescence Detection Process  Selection of Optimization Model  Selection of Optimization Model | S3  S3  S3 |

**Supplemental Figures**

| Figure S1. | The FL Intensity-potential Curves of the Biosensor for Multiple let-7 Family Members | S4 |
| --- | --- | --- |
| Figure S2.  Figure S3.  Figure S4.  Figure S5. | The Distinguishing of the Detachable DNA Assembly Module  Feasibility verification of miRNA-34 family analysis using detachable DNA assembly modules  Feasibility verification of miRNA-181 family analysis using detachable DNA assembly modules  Feasibility verification of microsatellite instability detection sites NR-21 using detachable DNA assembly modules | S4  S6  S7    S8 |
| Figure S6. | Assembly Schematic of the Ordered DNA Nanoflower | S9 |
| Figure S7.  Figure S8. | Characterization of DNA Nanoflower Structure and Biosensor Assembly  Performance evaluation of the assembly module for let-7a screening in complex samples. | S10  S10 |
| Figure S9. | Schematic Diagram of the Logic Gated Screening Route | S11 |
| Figure S10. | The Normalized Proportion of Each Experimental Group | S11 |
| Figure S11. | Specificity Analysis of let-7a in Cell Imaging Applications | S12 |
| Figure S12. | The Adhibition of the Detachable DNA Assembly Module in A549 cells | S13 |
| Figure S13.  Figure S14.  Figure S15.  Figure S16 | The Adhibition of the Detachable DNA Assembly Module in MCF-10A cells  The Adhibition of the Detachable DNA Assembly Module in hTERT-HPNE cells  The Adhibition of the Detachable DNA Assembly Module in PANC-1 cells  Dynamic monitoring of A549 cells under low-dose cisplatin treatment | S14  S15  S16  S18 |
| Figure S17.  Figure S18 | The Discrimination Verification of Detachable DNA Assembly Module to let-7b and let-7c  Machine learning algorithms for cells discrimination based on the next-generation the sequencing (NGS) | S19  S19 |
| Figure S119. | The independent analysis of individual concentration (IC) or individual proportion (IP) information for cells discrimination | S19 |
| Figure S20. | The ch index of K-means Clustering Classification Graph at the IC, IP, and IC-IP levels | S20 |

**Supplemental Tables**

| Table S1  Table S2 | DNA/RNA Sequences  The NGS- derived TFL and IC information | S20  S22 |
| --- | --- | --- |
| Table S3 | Distances between each case and the corresponding centroid  in the IC view | S22 |
| Table S4 | Distances between each case and the corresponding centroid  in the IP view | S22 |
| Table S5 | Distances between each case and the corresponding centroid  in the IC-IP view | S23 |

## Reagents

Duplex-specific nuclease (DSN) was purchased from Newborn (Shenzhen) Co., Ltd. Luminol (98%) was provided by LianGang Pigment and Dyestuff Chemical Industry Co., Ltd (Liaoning, China). H_2_O_2_ (30%) and amino-modified Fe_3_O_4_ magnetic bead (MB, 0.2-0.3 μm) were purchased from J&K Scientific Ltd. (Beijing, China). Hexanethiol (HT) was bought from Sigma Chemical Co. (St. Louis, MO, USA). N-(3-dimethylaminopropyl)-N′-ethylcarbodiimide hydrochloride (EDC) and N-hydroxy succinimide (NHS) were obtained from Shanghai Medpep Co. (Shanghai, China). Tris-(2-carboxyethyl)-phosphine hydrochloride (TCEP) was bought from Sangon Biotech Co., Ltd. (Shanghai, China). Gel green nucleic acid dye was obtained from Genview Scientific Inc. (Shanghai, China). Dopamine (DA) was purchased from Bide Pharmatech Ltd. (Shanghai, China). Hydrogen tetrachloroaurate (III) trihydrate (HAuCl_4_) was purchased from Sigma-Aldrich (St. Louis, MO, U.S.A). Trisodium citrate and exonuclease Ⅲ (Exo III) were purchased from Sangon Biotech (Shanghai) Co., Ltd.

Tris-HCl buffer solution (pH 7.4) was composed of 20 mM Tris, 1.0 mM MgCl_2_, 1.0 mM CaCl_2_, 140 mM NaCl, and 5 mM KCl for nucleotide sequence dilution. Tris-EDTA (TE, pH 8.0) buffer solution consists of 10 mM Tris-HCl, and 1.0 mM EDTA, which was used to dissolve and store oligonucleotides. The hybridization buffer (HB) was composed of 20 mM Tris-HCl, 20 mM MgCl_2_, and 140 mM NaCl for nucleotide sequence hybridization. Tris-magnesium salt (TM, pH 8.0) buffer solution was composed of 10 mM Tris-HCl, and 50 mM MgCl_2_ for self-assembly of ordered DNA nanoflower. The phosphate-buffered saline (PBS, pH 7.4) used in the experiment was composed of 100 mM Na_2_HPO_4_, 100 mM KH_2_PO_4_, and 100 mM KCl to test the performance of the biosensor. 5×Tris-boric acid-EDTA buffer (TBE buffer, pH 8.0) was composed of 445 mM Tris base, 445 mM boric acid, and 10 mM EDTA for the polyacrylamide gel electrophoresis (PAGE) experiments. The water used in the experiment was ultra-pure. All the nucleic acid chains used in this work were purified by high-performance liquid chromatography and purchased from Sangon Biotech (Shanghai) Co., Ltd.

## Apparatus

The fluorescence signal responses were recorded by the F-7000 fluorescence spectrophotometer (Hitachi, Tokyo, Japan). Native polyacrylamide gel electrophoresis was performed with a BG-verMIDI standard vertical electrophoresis apparatus (Baygene, China) and a Gel Doc XR+ System (Bio-Rad, California, U.S.A.). Atomic force microscope (AFM) was carried on a multimode 8 microscope (Bruker, Germany). The electrochemiluminescence (ECL) measurements were recorded on an MPI-A multifunctional analyzer (Xi'An Remax Electronic Science & Technology Co. Ltd., Xi'An, China). Cyclic voltammetry (CV) was accomplished by a CHI760E electrochemical workstation (CH Instruments, Shanghai, China) with a three-electrode arrangement. Fluorescence imaging of living cells was used by an Olympus IX-81 microscope (Olympus, Japan). A PHS-3C digital pH meter (Shanghai LeiCi Device Works, Shanghai, China) was employed to test the pH of all solutions in the whole experiment.

## Cells Culture and Lysate Extract

The human lung carcinoma epithelial cells (A549 cells) and human breast cancer cells (MCF-7 cells) were maintained in a DMEM medium containing 1% non-essential amino acids, 100 U/mL penicillin, and 10% fetal bovine serum (FBS). The human normal mammary epithelial cells (MCF-10A) were cultured in a specialized and complete MCF-10A medium. All cells were cultivated at 37°C in a humidified atmosphere comprised of 95% air and 5% CO_2_. After 24 hours of incubation, 1×10^6^ cells in the exponential phase were collected and then washed two times with sterile PBS. Total RNA extraction for real sample detection was performed using the Trizol Reagent Kit (Sangon, Inc., Shanghai, China) following the manufacturer's instructions. Finally, the cellular extracts were diluted and stored at -80°C to prepare them for future use.

## Electrochemiluminescence Detection Process

First, different concentrations of let-7a were respectively added to the mixture containing 20 μL 10×Exo Ⅲ buffer and 2 μL Exo III (400 U) to generate the sample solution. After obtaining the initialized ECL signal of the biosensor in the absence of let-7a, the biosensor was incubated with 10 μL of sample solution at 37℃ for 2 h. PBS (0.1 M, pH 7.4) was used to rinse and remove the unbonded residue. Finally, the biosensor was immersed in 2 mL PBS (0.1 M, pH 7.4) containing 15 μM luminol to record the ECL signal. The ECL detection parameters were set as follows: the high voltage of the photomultiplier tube was 800 V, the scanning speed was 300 mV/s, and the voltage range was -0.6 V to 0.6 V.

## Cellular fluorescence imaging

A549 cells, MCF-7 cells and MCF-10A cells were introduced into a 35 mm^2^ Petri dish. They were respectively cultured in DMEM medium and commercial MCF-10A specific culture medium with a humidified atmosphere (5% CO_2_) immediately, at 37℃ for 24 h to reach 80% cell anchorage-dependent rate, and then washed with sterile 1×PBS. Next, Add the corresponding DNA/RNA (1 uM), DSN (1 U) and 0.2 x DSN buffer with medium. When the cell density reached 80% of the maximum adherent growth density, 95 μL of samples were respectively mixed with 5 μL lipofectamine 3000 and added into the culture dishes. After incubated 6 h, the cells were stained with 70 μL Hoechst 33342 solution for 15 min and then washed 3 times with 1× PBS. After washing, the cells were incubated with 1 mL of fresh DMEM medium at 37 °C before fluorescence imaging.

## Selection of Optimization Model

Firstly, the let-7 family exhibits high homogeneity, which means that there is a small variation in the base sequences among its family members. Then, considering that most members of the let-7 family have no more than two base differences from let-7a, and based on the DIANA-miTED database^1^, it can be determined that these family members account for the vast majority (over 90%) of the total family level. Hence, we approximated the expression level of these family members to the total level of the let-7 family. Meanwhile, due to the largest nucleotide difference between let-7b and let-7a within the above members, let-7b was chosen as the validation target during the stem optimization to ensure that these let-7 family members can participate widely in module assembly.

## Continuous State Transition of the Detachable DNA Assembly Module


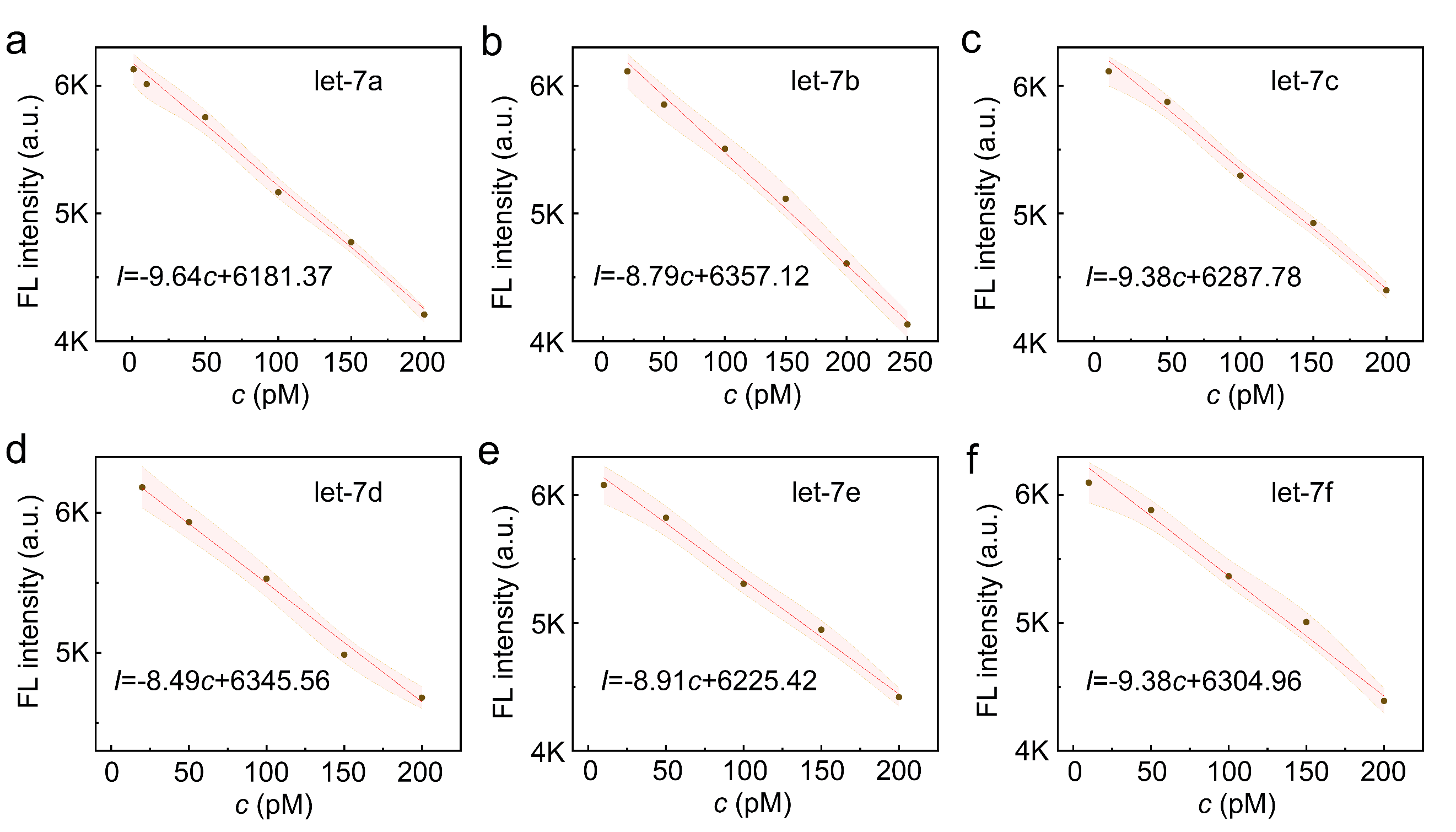


**Figure S1.** The FL intensity-potential curves of the biosensor incubated with different concentrations of (a) let-7a, (b) let-7b, (c) let-7c, (d) let-7d, (e) let-7e, and (f) let-7f.


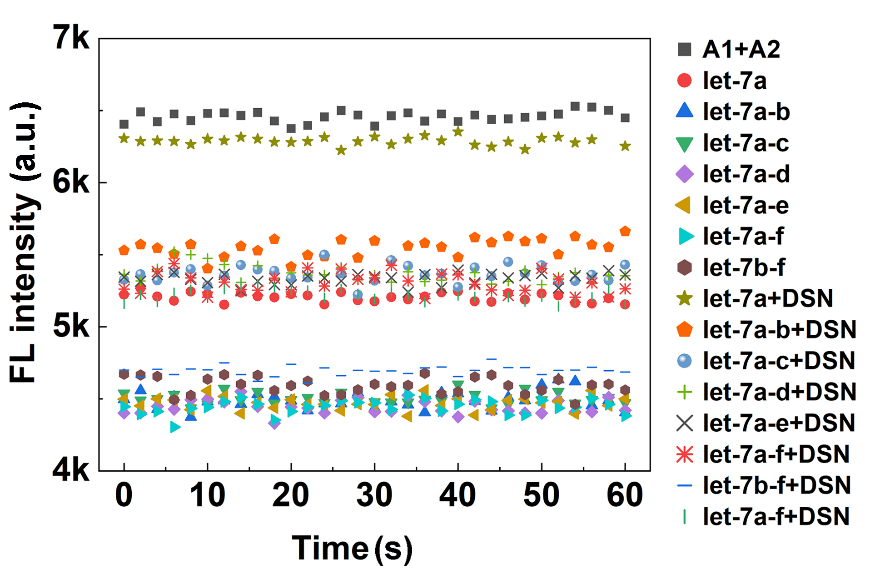


**Figure S2.** The distinguishing of detachable DNA assembly module to let-7a in a mixture of family members. The concentration of let-7a was constant at 10 nM, and other family members totaled 10 nM in the same proportion.

Building on the validation of the detachable DNA assembly module's applicability to the let-7 family, we further explored its suitability for other RNA families. Here, we selected two additional highly homologous RNA families: the miRNA-34 family and the miRNA-181 family. First, we validated the applicability of the detachable DNA assembly module to the miRNA-34 family. Using miRNA-34a as the target RNA, we executed performance validation through gel electrophoresis analysis and fluorescence response analysis. As shown in **Figure** S3a, we first verified the participation of miRNA-34a in module assembly and subsequent module disassembly. The results indicated that when miRNA-34a was present, a significantly slower migrating band appeared, suggesting successful module assembly. Furthermore, when this product was treated with DSN, it was successfully disassembled, implying the disassembly of the module. Additionally, the fluorescence response analysis revealed that a strong fluorescence quenching occurred when miRNA-34a was added to the system, and then the existence of DSN strongly revived the signal (Figure S3b). This result fully demonstrated that miRNA-34a could participate in both the assembly and disassembly of the module.

Furthermore, we validated the ability of the detachable DNA assembly module to distinguish miRNA-34a from other family members. As shown in Figure S3c, all miRNA-34 family members could participate in module assembly. However, after treatment with DSN, only the module assembled with miRNA-34a was successfully disassembled, indicating that the detachable DNA assembly module has excellent specificity for miRNA-34a. To further confirm this conclusion, we also conducted fluorescence response analysis. As expected, the addition of each miRNA-34 family member efficiently quenched the signal, but only the experimental group with miRNA-34a achieved subsequent signal recovery (Figure S3d). Therefore, these experiments demonstrated that the detachable DNA assembly module has the ability to recognize the miRNA-34 family and efficiently screen for the target miRNA-34a.

Additionally, we demonstrated the applicability of the detachable DNA assembly module to the miRNA-181 family using the same approach (Figure S4). These results indicate that the proposed detachable DNA assembly module possesses versatility for multi-target detection.


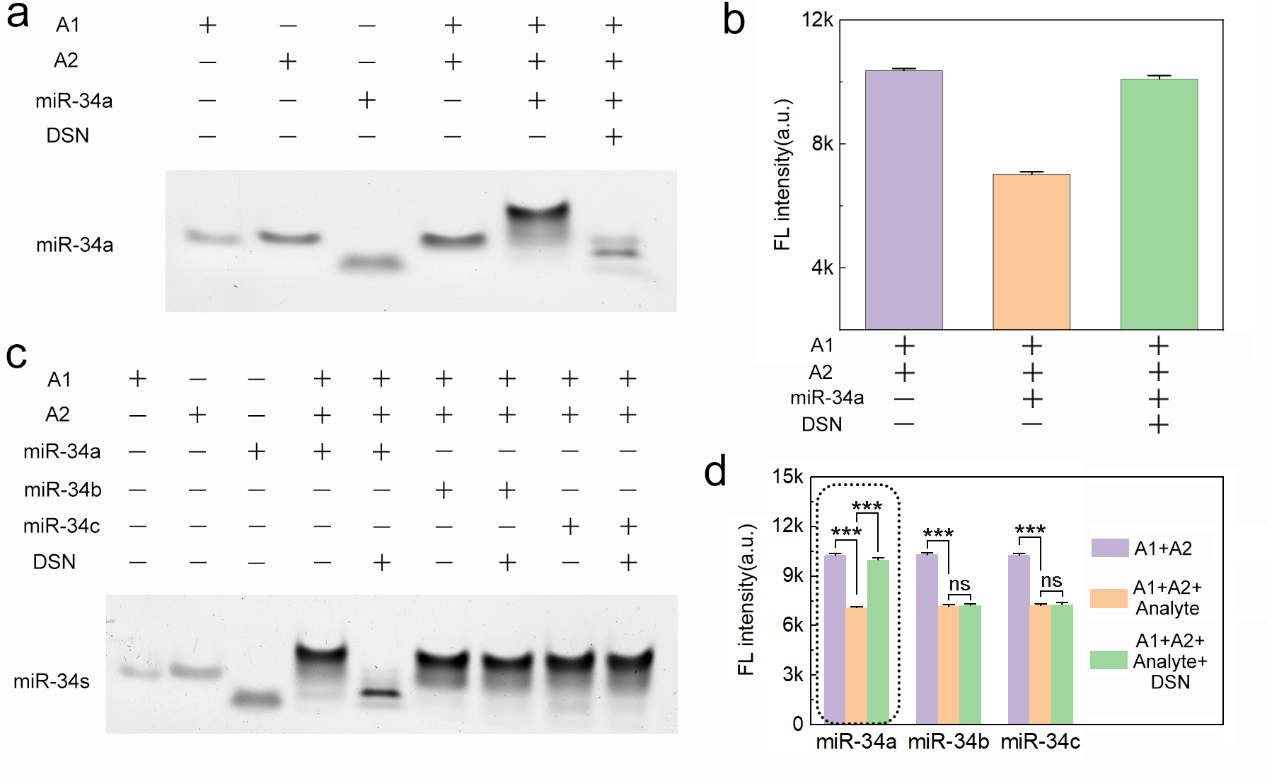


**Figure S3**. Feasibility verification of miRNA-34 family analysis using detachable DNA assembly modules. a) Gel electrophoresis characterization and b) fluorescence response analysis of continuous response of detachable DNA assembly module to single miRNA-34a (miR-34a). c) Gel electrophoresis characterization and d) fluorescence response analysis of continuous response of detachable DNA assembly module to miRNA-34 family members (miR-34s).


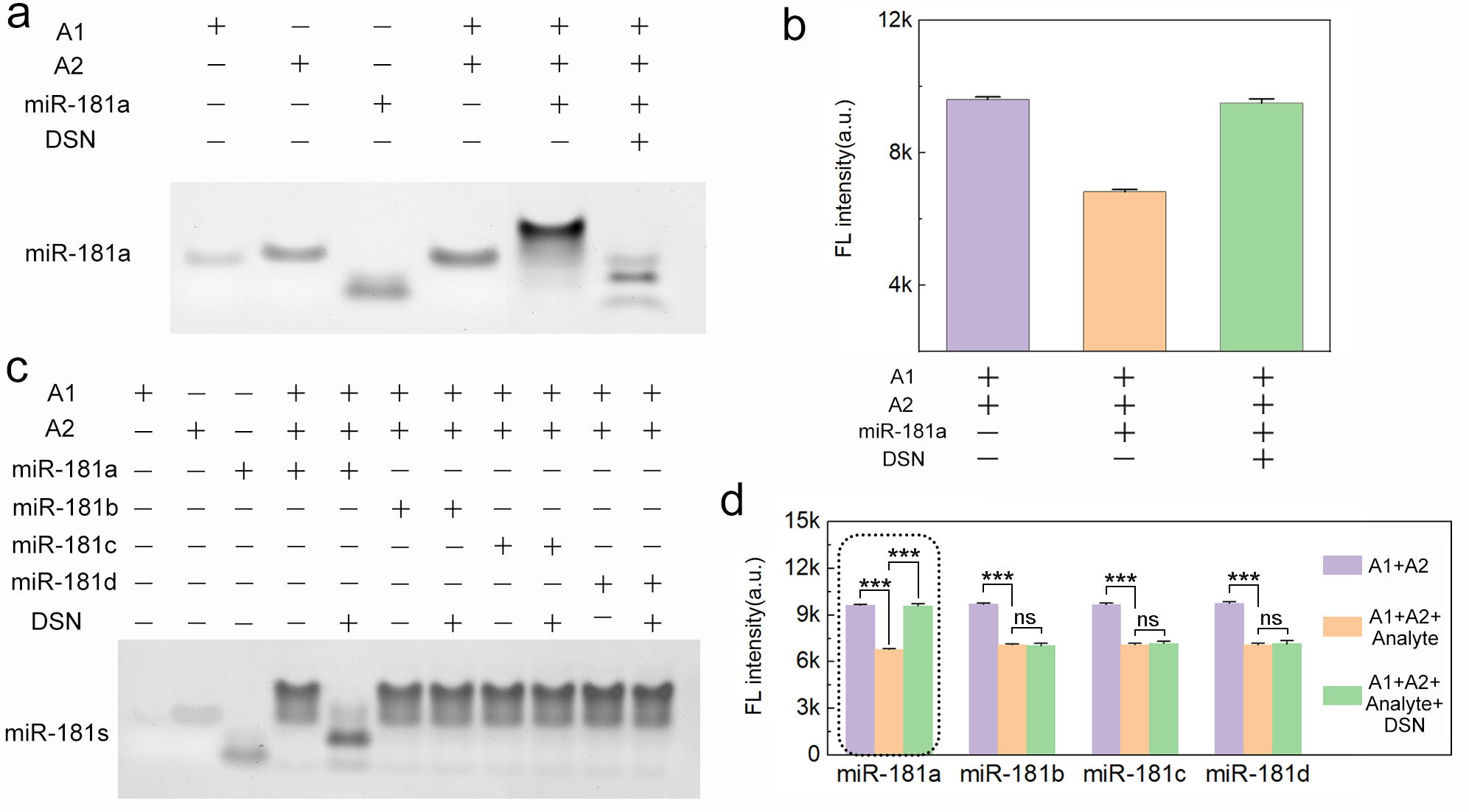


**Figure S4**. Feasibility verification of miRNA-181 family analysis using detachable DNA assembly modules. a) Gel electrophoresis characterization and b) fluorescence response analysis of continuous response of detachable DNA assembly module to single miRNA-181a (miR-181a). c) Gel electrophoresis characterization and d) fluorescence response analysis of continuous response of detachable DNA assembly module to miRNA-181 family members (miR-181s).

To explore the potential application of the proposed detachable DNA assembly module in microsatellite instability analysis, we selected the classic single nucleotide repeat site NR-21 as a validation model. First, we simulated several instability scenarios of NR-21, namely base insertion (NR-21-I) or deletion (NR-21-D), as well as additional base mutation (NR-21-M). As shown in Figure S5a, we used NR-21 as the target to investigate the feasibility of continuous response by the detachable DNA assembly module. When NR-21 was present, a significantly retarded band appeared, and treatment with DSN further efficiently disassembled this structure, indicating that NR-21 could participate in both module assembly and disassembly. Meanwhile, fluorescence response analysis corroborated these conclusions (Figure S5b).

On this basis, we further validated the ability of the detachable DNA assembly module to distinguish NR-21 from its instability variants. As shown in Figure S5c, sequences under NR-21 instability conditions effectively promoted module assembly, facilitating effective tracking of these conditions. Subsequently, DSN only disassembled the module assembled with NR-21, demonstrating the high specificity of the detachable DNA assembly module for NR-21. Additionally, fluorescence response analysis showed that only the NR-21 experimental group achieved continuous fluorescence quenching and recovery, while other experimental groups only exhibited fluorescence quenching (Figure S5d). These results indicate that the detachable DNA assembly module can efficiently recognize and screen NR-21, proving its potential application in other microsatellite instability analyses.


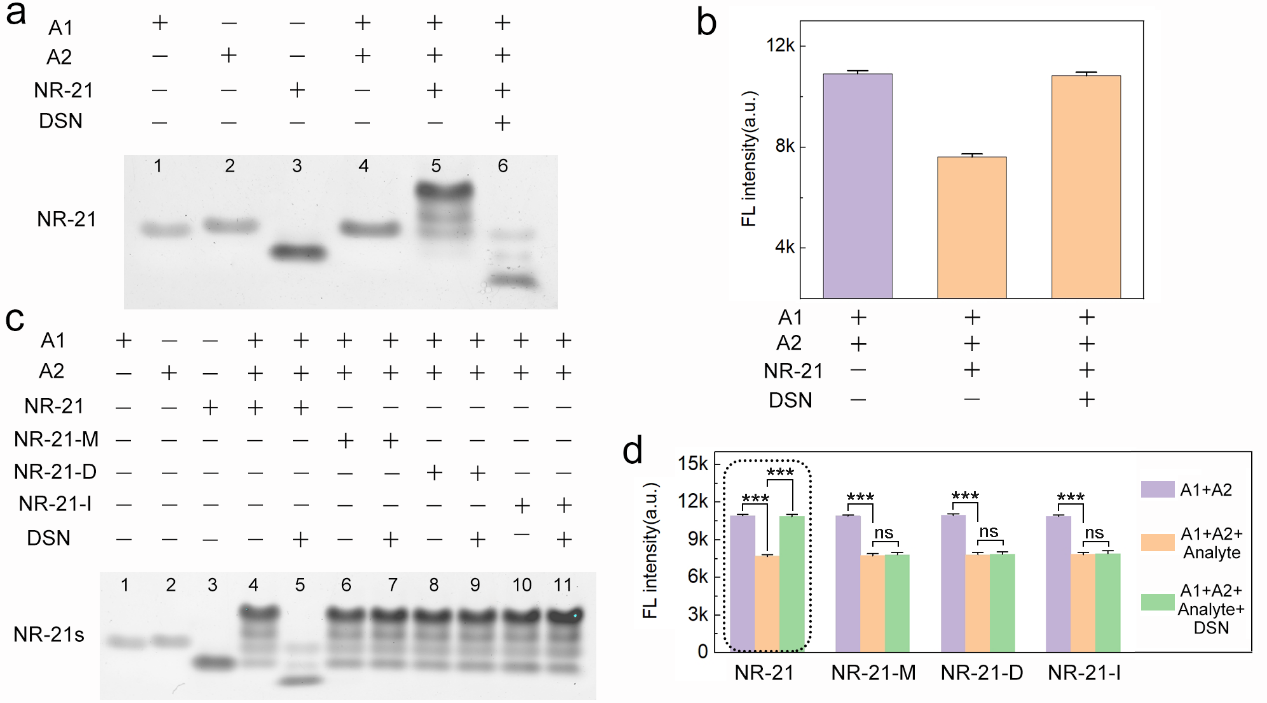


**Figure** **S5**. Analysis feasibility verification of microsatellite instability detection sites NR-21 using detachable DNA assembly modules. a) Gel electrophoresis characterization and b) fluorescence response analysis of continuous response of detachable DNA assembly module to NR-21. c) Gel electrophoresis characterization and d) fluorescence response analysis of continuous response of detachable DNA assembly module to NR-21M, NR-21D, and NR-21I.

## Proposal and Validation of Evaluation Tools

The preparation process of ordered DNA nanoflower was diagrammatized in Figure S6. Firstly, Sulfo-SMCC (200 mM) was added to the dopamine (DA) solution at room temperature for 2 h. After that, thiol-modified B1, B2, and B3 were treated with 1 M TCEP at room temperature for 30 min, respectively. Then the resultant solution was mixed with DA solution for 2 h to generate DA-modified DA-B1, DA-B2, and DA-B3, respectively. Finally, DA-B1, DA-B2, DA-B3, B4, blocking chain, and capture probe (CP) were mixed and annealed at 95℃ for 10 min, and then quickly transferred to the refrigerator at 4℃ to form ordered DNA nanoflower.


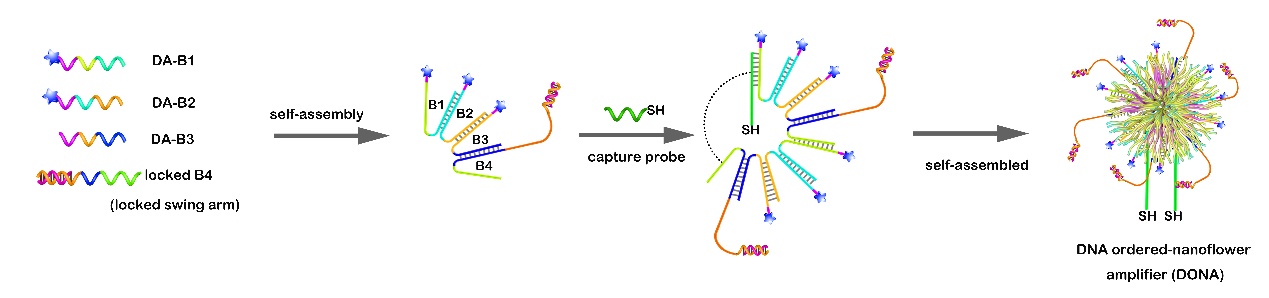


**Figure S6.** Schematic diagram of the assembly of the ordered DNA nanoflower.

The electrochemiluminescence behaviors of modified GCE were studied to verify the assembly process of GCE. As shown in **Figure** S7a, bare GCE exhibited an obvious ECL signal (curve a) due to the presence of luminol. When Au NPs were modified on the surface of GCE (AuNPs/GCE), the ECL signal increased due to the promotion of AuNPs to electron transport of the luminol/dissolved oxygen system (curve b). When the DNA nanoflower was modified on the AuNPs/GCE surface by the Au-S bond, the ECL signal of the resultant GCE named DFs/AuNPs/GCE decreased significantly (curve c). Curve d showed that the response of ECL decreased ulteriorly after the incubation of HT (HT/DFs/AuNPs/GCE) due to the electron transfer hindrance of HT^3^.

As shown in Figure S7b, modified GCEs were immersed in [Fe(CN)_6_]^3-/4-^ (5 mM) containing 0.1 M KCl to investigate the current response. The bare GCE showed a pair of standard CV peaks (curve a). When GCE was modified with Au NPs, the CV current value of the modified electrode increased (curve b). The ordered DNA nanoflower was a nucleic acid macromolecular complex, which greatly resisted the diffusion of the redox substance [Fe(CN)_6_]^3-/4-^ on the electrode surface, so when the ordered DNA nanoflower was modified on AuNPs/GCE, the CV value decreased obviously (curve c). When HT was used to block the non-specific binding site of the GCE, the CV current of the electrode decreased again (HT/DFs/AuNPs/GCE). The electrochemical behavior of the above electrode surface proved that the biosensor was successfully constructed.


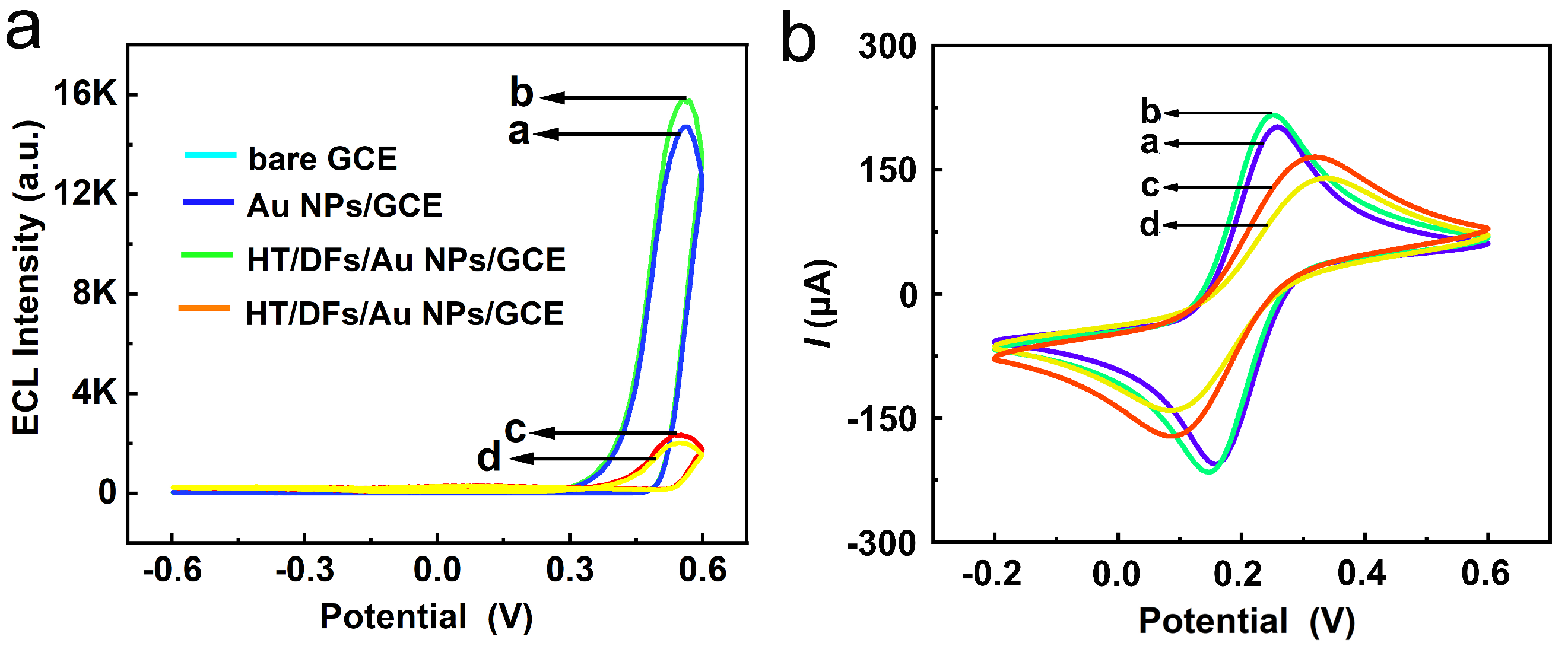


**Figure S7.** Performance analysis of DNA nanoflower-based biosensors as evaluation tools. a) ECL responses of the GCE with different modifications in 2 mL PBS (pH 7.4) containing 15 μM luminol. b) CV waves of the different electrodes in PBS (pH = 7.4) containing 5.0 mM [Fe(CN)_6_]^3−/4−^. Bare GCE (curve a), AuNPs/GCE (curve b), ordered DNA nanoflower/Au NPs/GCE (curve c), HT/DFs/Au NPs/GCE (curve d).

## Performance Evaluation of the Assembly Module for let-7a Screening in Complex Samples


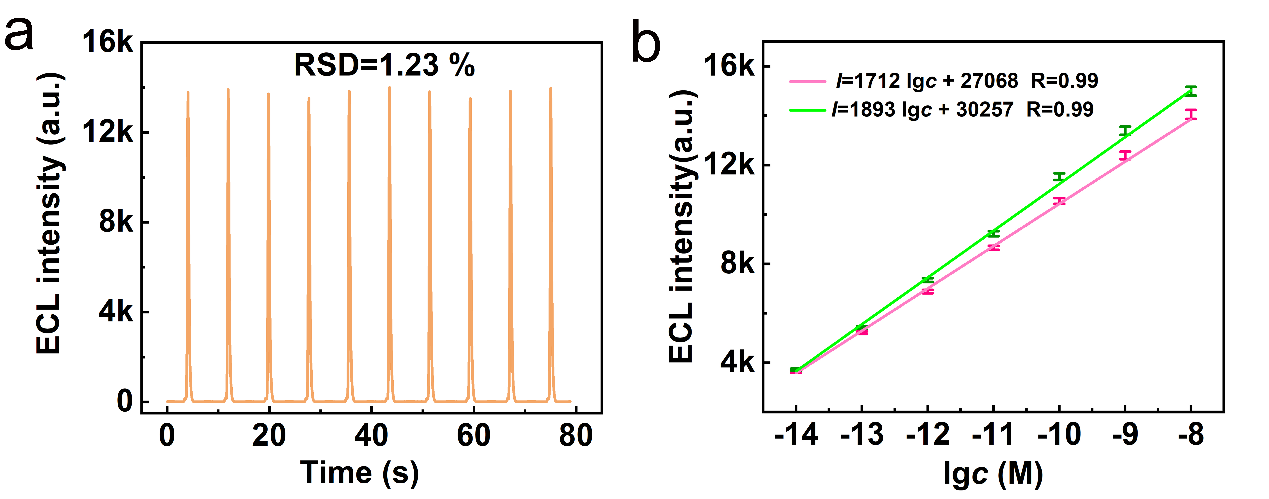


**Figure S8.** Performance evaluation of the assembly module for let-7a screening in complex samples**.** a) The stability of the combined mode for 10 nM let-7a. b) The fitted linear response curve of ECL characterization.

## Intracellular Imaging Applications of Detachable DNA Assembly Module


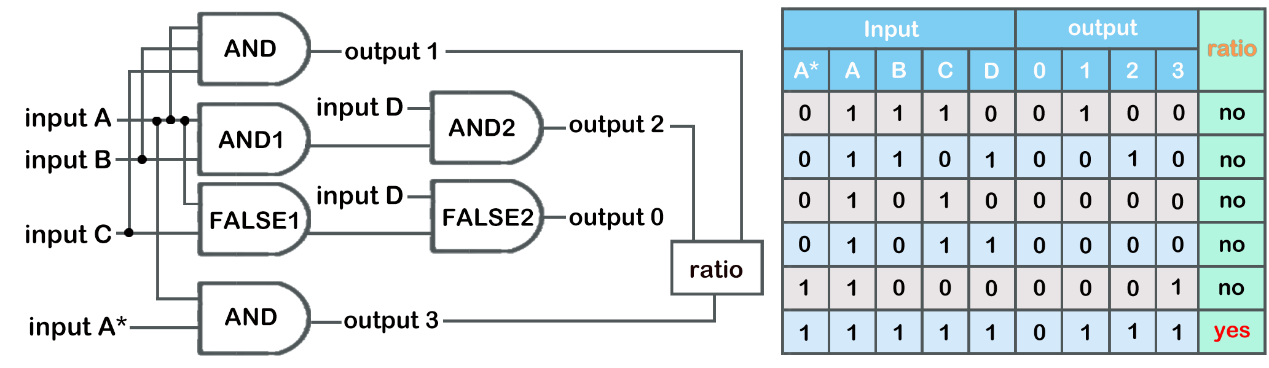


**Figure S9.** Schematic diagram of the logic gate screening route.


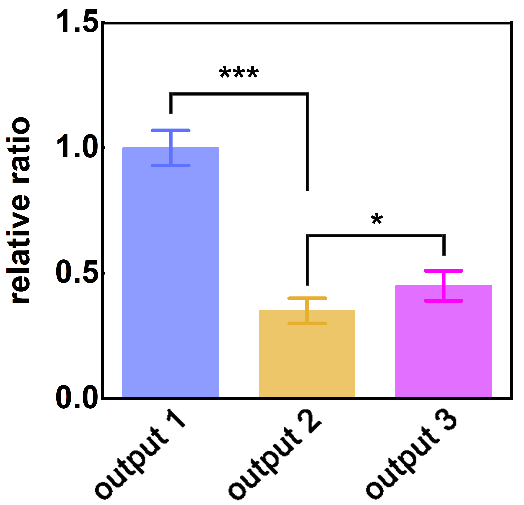


**Figure S10.** The normalized proportion of each experimental group. ^*^p<0.05, and ^***^p<0.001 in t-test.

As shown in Figure S11, the silence chain caused fluorescence enhancement compared to the control group in which MCF-7 cells were processed by input A alone. However, when DSN was introduced, the signal of input A-treated MCF-7 cells increased significantly, while the silent chain-treated MCF-7 cells were still in a state of fluorescence quenching, indicating that the output variation was induced by the disintegration of let-7a-involved detachable DNA assembly module. Furthermore, after the introduction of let-7a analogs based on the silent chain, the output was still weak after incubation with DSN, which is due to the high sensitivity of DSN to mismatch events.


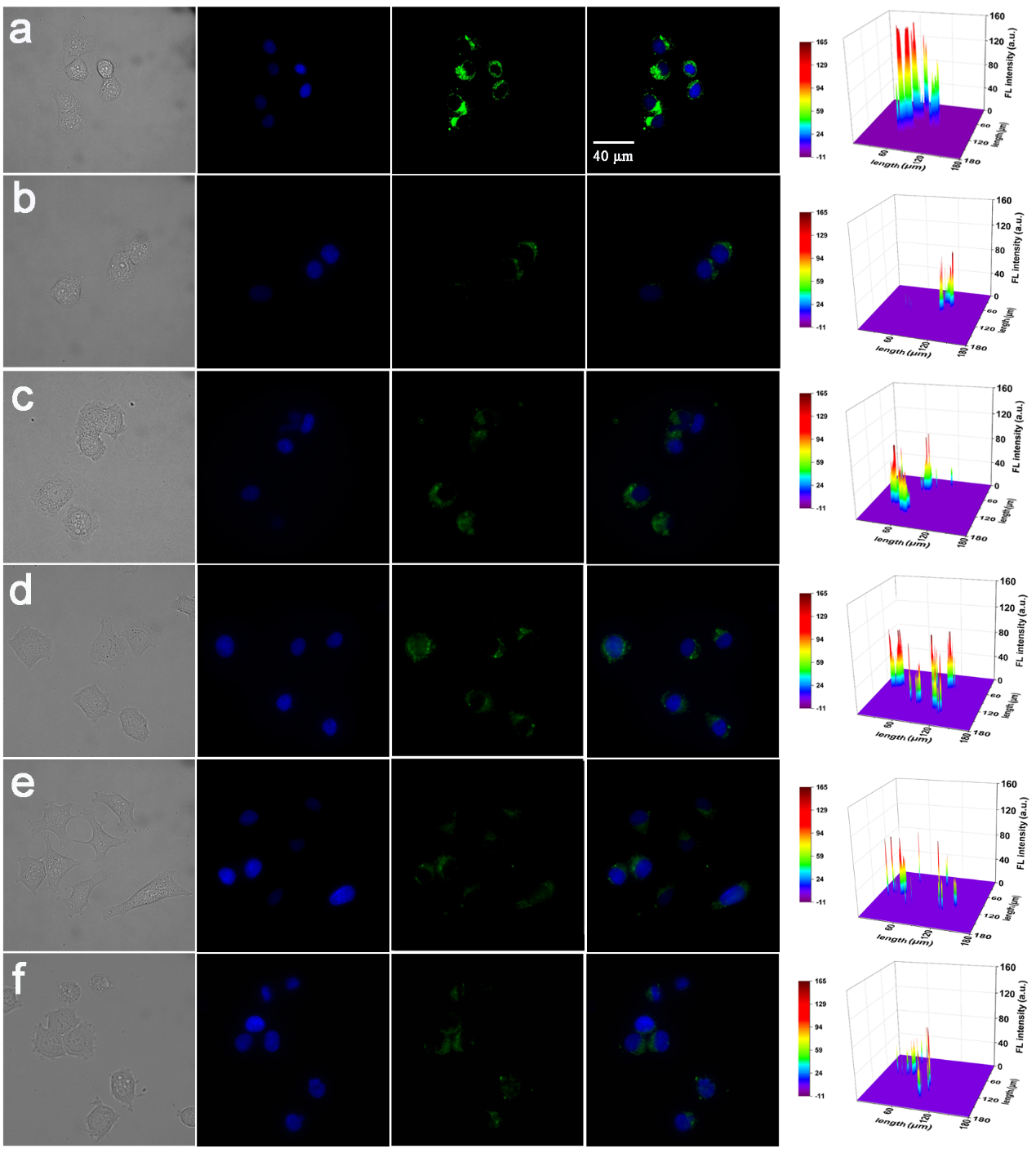


**Figure S11.** Fluorescence images of MCF-7 with different treatment processes. a) input A&A^*^, (b) input A, (c) silence chain of let-7a and input A, (d) input A and DSN, (e) silence chain of let-7a, input A, and DSN, (f) silence chain of let-7a, analog, input A, and DSN. Scale bar = 40 μm.


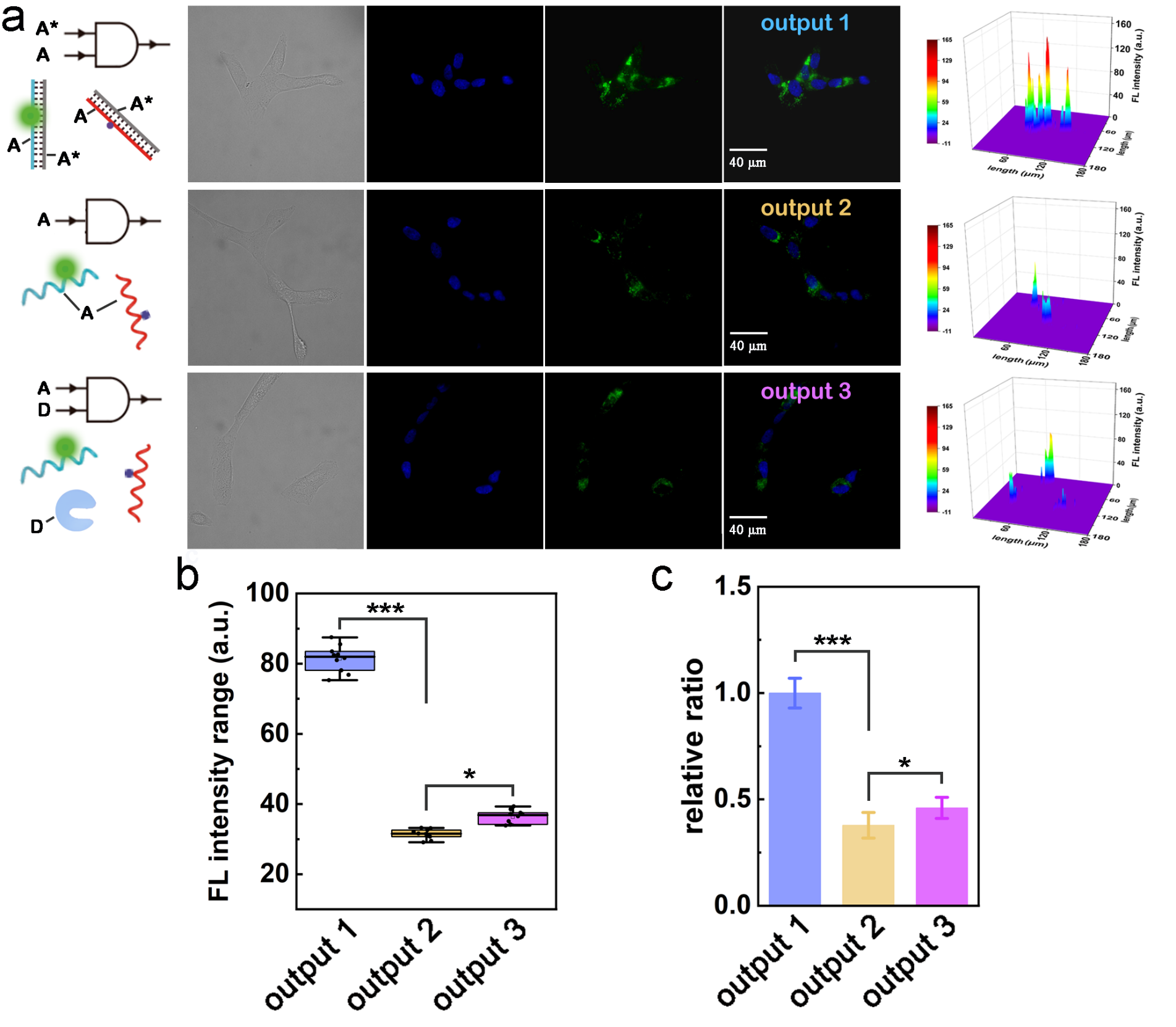


**Figure S12.** Application of the detachable DNA assembly module in A549 cells. a) Fluorescence images of A549 cells incubated with input A alone, both of input A and input D, and both of A and A^*^and (b) average fluorescence intensity of quantitative statistics. c) Fluorescence output range of A549 cells treated in different experimental groups and (d) the normalized proportion of each experimental group. ^*^*p*<0.05, and ^***^*p*<0.001 in *t*-test.


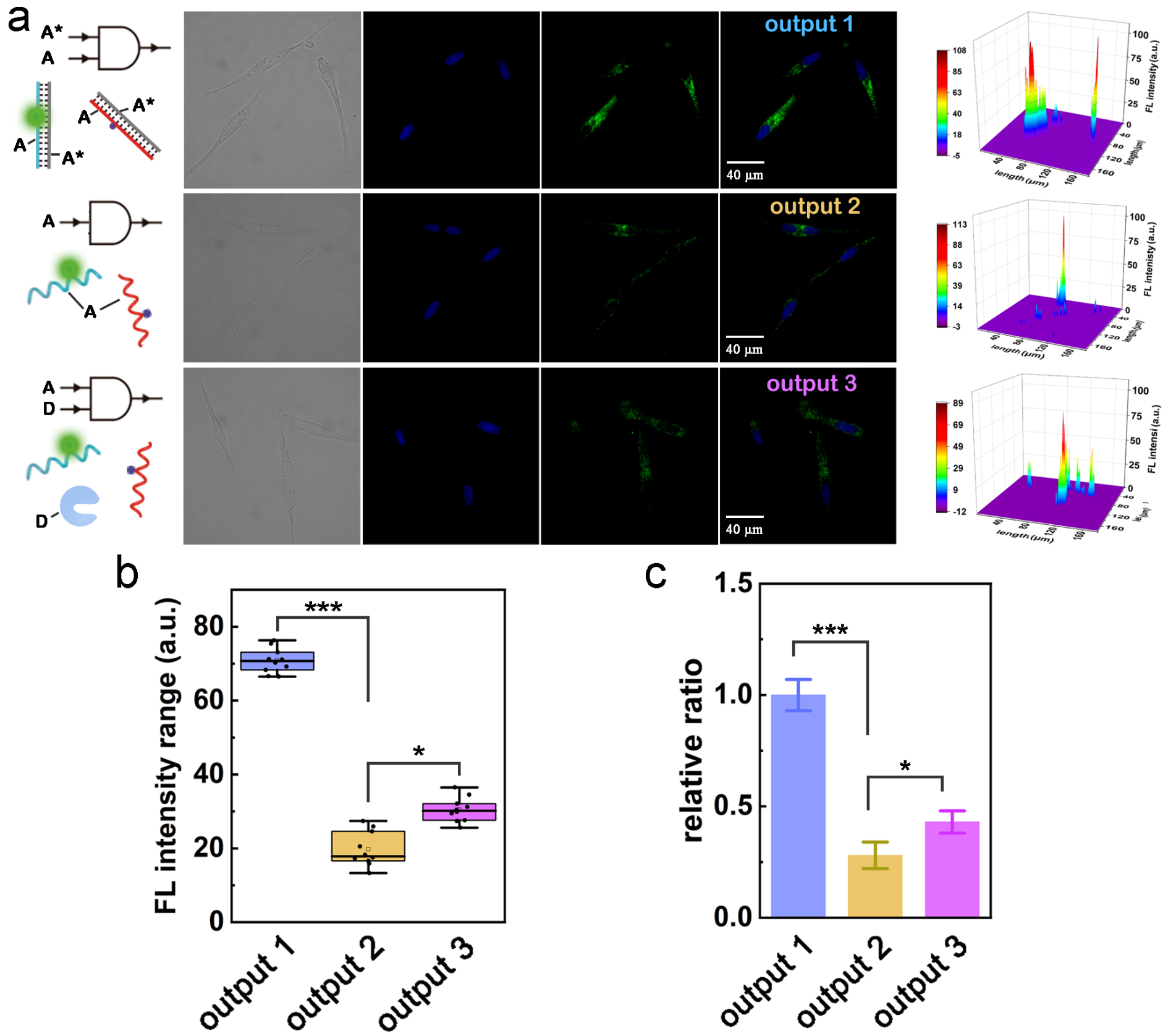


**Figure S13.** Application of the detachable DNA assembly module in MCF-10A cells. a) Fluorescence images of MCF-10A cells incubated with input A alone, both of input A and input D, and both of A and A^*^and (b) average fluorescence intensity of quantitative statistics. c) Fluorescence output range of MCF-10A cells treated in different experimental groups and (d) the normalized proportion of each experimental group. ^*^*p*<0.05, and ^***^*p*<0.001 in *t*-test.


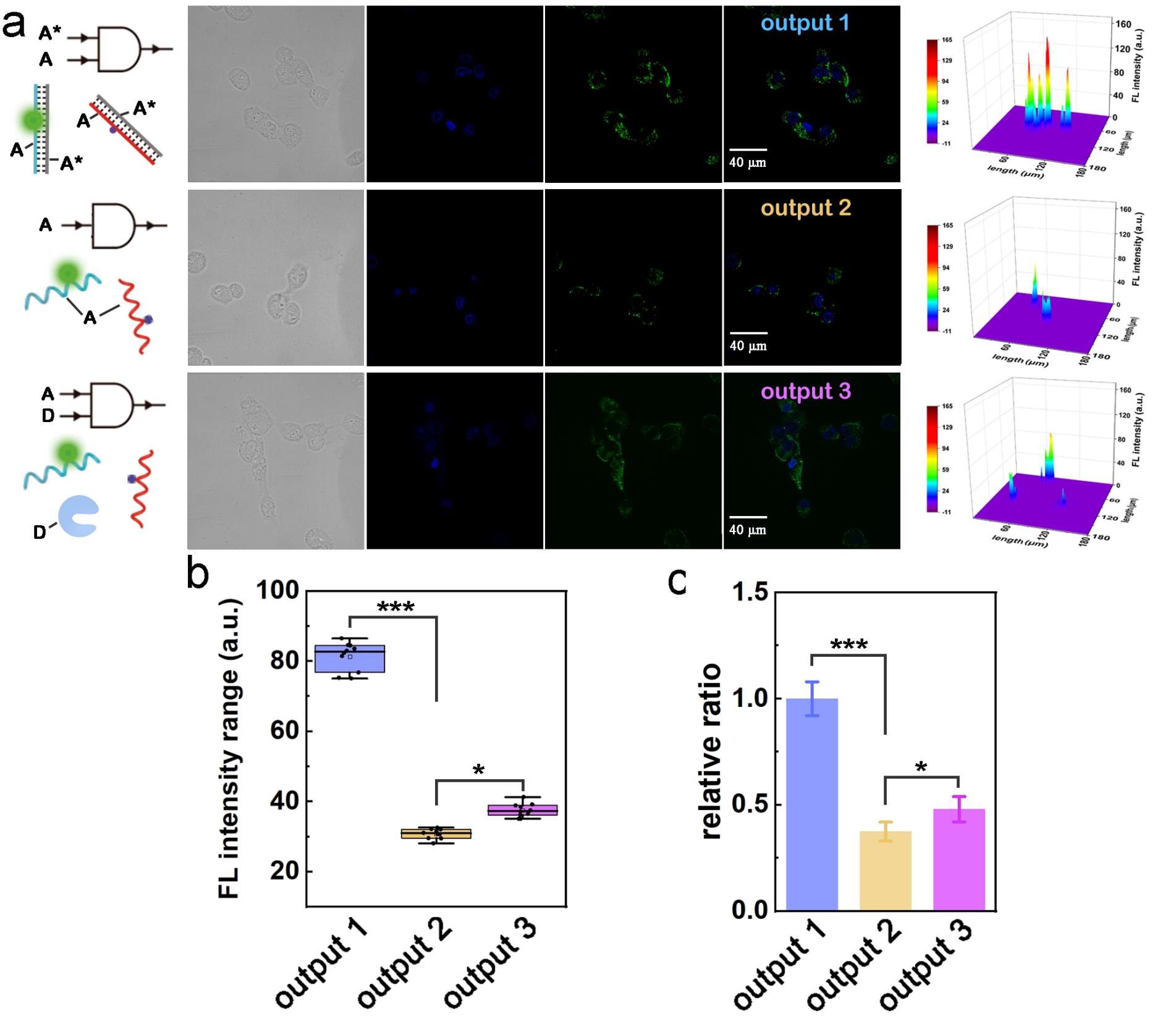


**Figure S14.** Application of the detachable DNA assembly module in hTERT-HPNE cells. a) Fluorescence images of hTERT-HPNE cells incubated with input A alone, both of input A and input D, and both of A and A^*^and (b) average fluorescence intensity of quantitative statistics. c) Fluorescence output range of hTERT-HPNE cells treated in different experimental groups and (d) the normalized proportion of each experimental group. ^*^*p*<0.05, and ^***^*p*<0.001 in *t*-test.


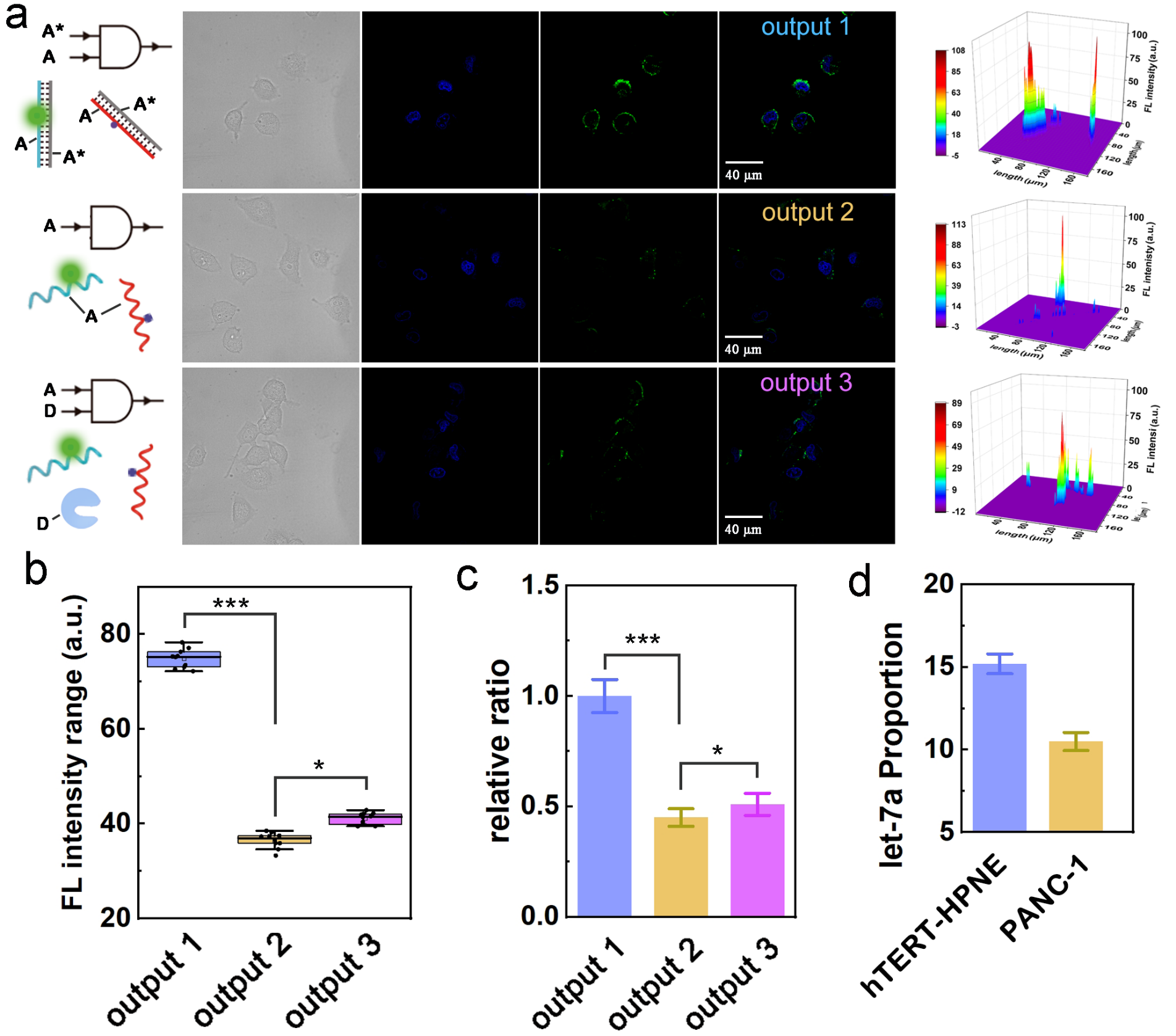


**Figure S15.** Application of the detachable DNA assembly module in PANC-1 cells. a) Fluorescence images of PANC-1 cells incubated with input A alone, both of input A and input D, and both of A and A^*^and (b) average fluorescence intensity of quantitative statistics. c) Fluorescence output range of PANC-1 cells treated in different experimental groups and (d) the normalized proportion of each experimental group. ^*^*p*<0.05, and ^***^*p*<0.001 in *t*-test.

Considering the high screening ability of the detachable DNA assembly module for specific RNA sequences in highly homologous RNA families and current research reports that cisplatin, as a chemotherapeutic agent, upregulates the expression of let-7a in A549 cells.^4-6^ We applied the detachable DNA assembly module to further dynamically monitor the effects of the cisplatin on let-7 family and let-7a proportion in A549 cells. The general analysis process was as follows: First, the module components A1 and A2 as input A was blocked with complementary strands (input A*) to prevent them from completing modular assembly with the let-7 family in A549 cells. Subsequently, we treated a new batch of A549 cells with a low concentration of cisplatin (2 μM). Immediately after the treatment of cisplatin, we tracked the cell status over different time intervals. Then, at various time points, the cells were treated sequentially with (1) module components input A and (2) DSN enzyme to obtain fluorescence signals. Finally, by statistically analyzing the fluorescence intensity from imaging, the fluctuation of let-7a levels with different durations of cisplatin treatment could be estimated.

As shown in Figure S16a, the blocked input A (input A&A*) obtained a strong fluorescence signal as the initial signal. After treated a new batch of A549 cells with a low concentration of cisplatin (2 μM), the fluorescence signal continuously decreased when input A was transfected, with a rapid decline observed between 12 and 24 hours. This indicated a rapid response of A549 cells to cisplatin stress and damage repair mechanisms, leading to upregulation of the let-7 family. With the increase of cisplatin treatment time, the difference in fluorescence recovery before and after DSN enzyme (input D) transfection became more pronounced, validating the upregulation of let-7a expression. The quantification of fluorescence intensity under input A and input D transfections was shown in Figures S16b and S16c, respectively. By comparing fluorescence quenching due to input A and fluorescence recovery due to input D at different time points, we inferred the proportion of let-7a at each time interval. As depicted in Figure S16d, within 6-24 hours of cisplatin treatment, the proportion of let-7a significantly increased, and subsequently slowed in 24-36 hours. These results indicated that within a short duration of cisplatin treatment, although there is no substantial apoptosis, the cells have already initiated a strong and sustained stress and damage repair response. The cumulative effects of these responses eventually lead to cell apoptosis.


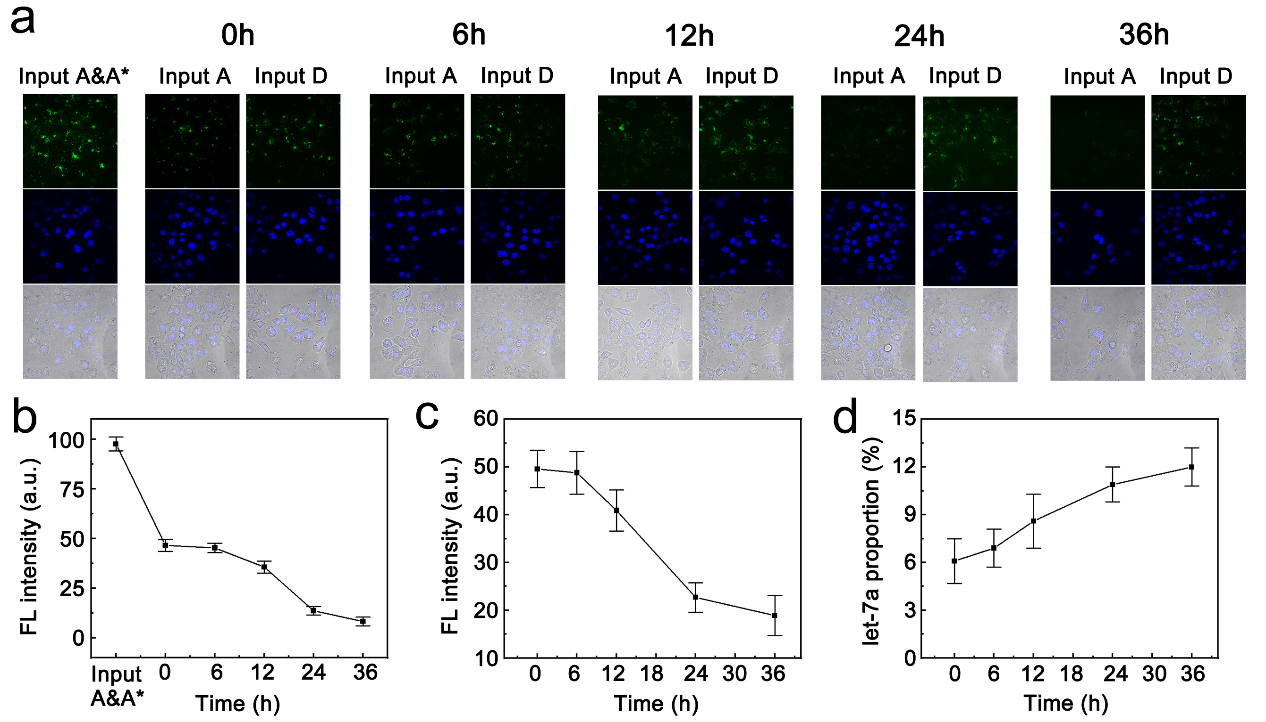


**Figure S16**. Dynamic monitoring of A549 cells under low-dose cisplatin treatment by the detachable DNA assembly module. a) Dynamic monitoring of A549 cells under low-dose cisplatin treatment using a detachable DNA assembly module at different time points (0 h, 6 h, 12 h, 24 h, and 36 h). b, c) Grayscale quantification analysis of A549 cell fluorescence images under (b) input A and (c) input D treatments at different time points. d) The proportion of let-7a calculated at different time points.

## Detachable DNA Assembly Module for Cells Discrimination

As shown in **Figure** S17a, when Module-b was used for the analysis of multiple let-7 family members, all modules exhibited significant and similar fluorescence quenching before DSN enzyme treatment, indicating that Module-b possessed the property of co-capture to multiple family members. After the DSN enzyme was added to the system, only the fluorescence signal of the let-7b experimental group significantly recovered, while the signal of the other experimental groups remained almost unchanged. The above results fully demonstrated the feasibility of Module-b for screening let-7b. Using the same principle, Module-c also confirmed its specificity for let-7c (Figure S17b).


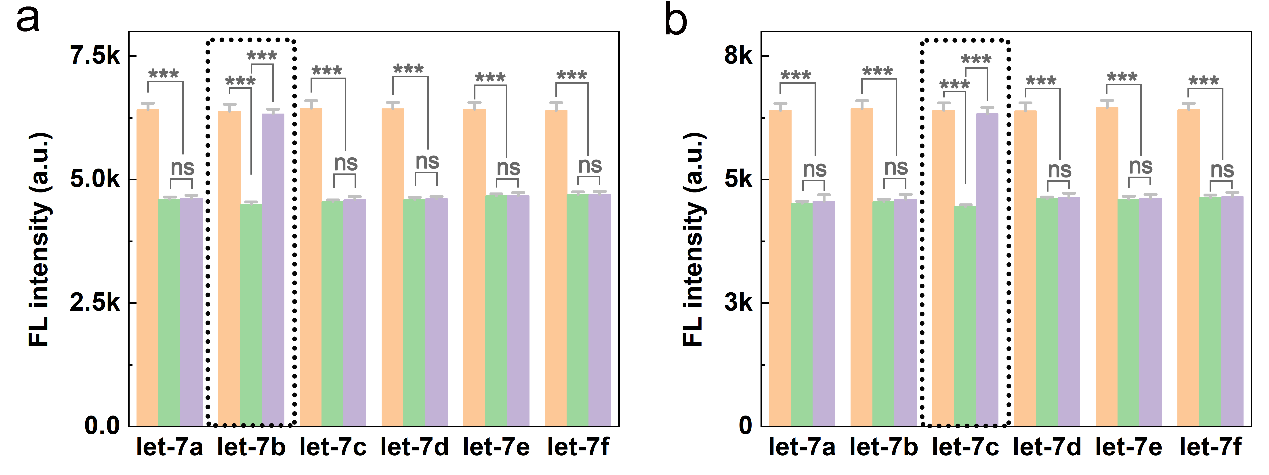


**Figure S17.** The discrimination verification of the detachable DNA assembly module to (a) let-7b and (b) let-7c. The concentration of analyte in each experimental group was 20 nM. orange means A1+A2; green means A1+A2+target RNA; violet means A1+A2+analyte+DSN. ^***^*p*<0.001; ns, non-significant in *t*-test.


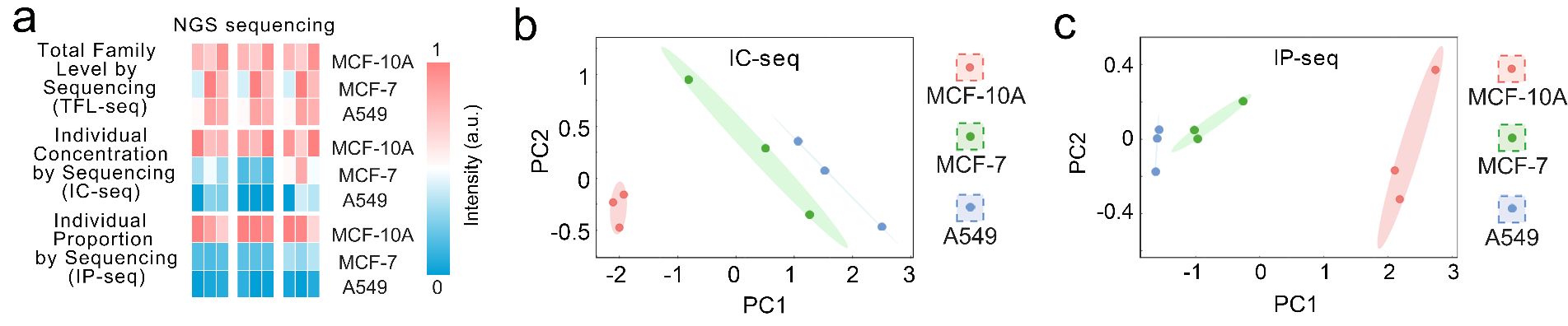


**Figure S18.** Machine learning algorithms for cells discrimination based on the next-generation the sequencing (NGS).


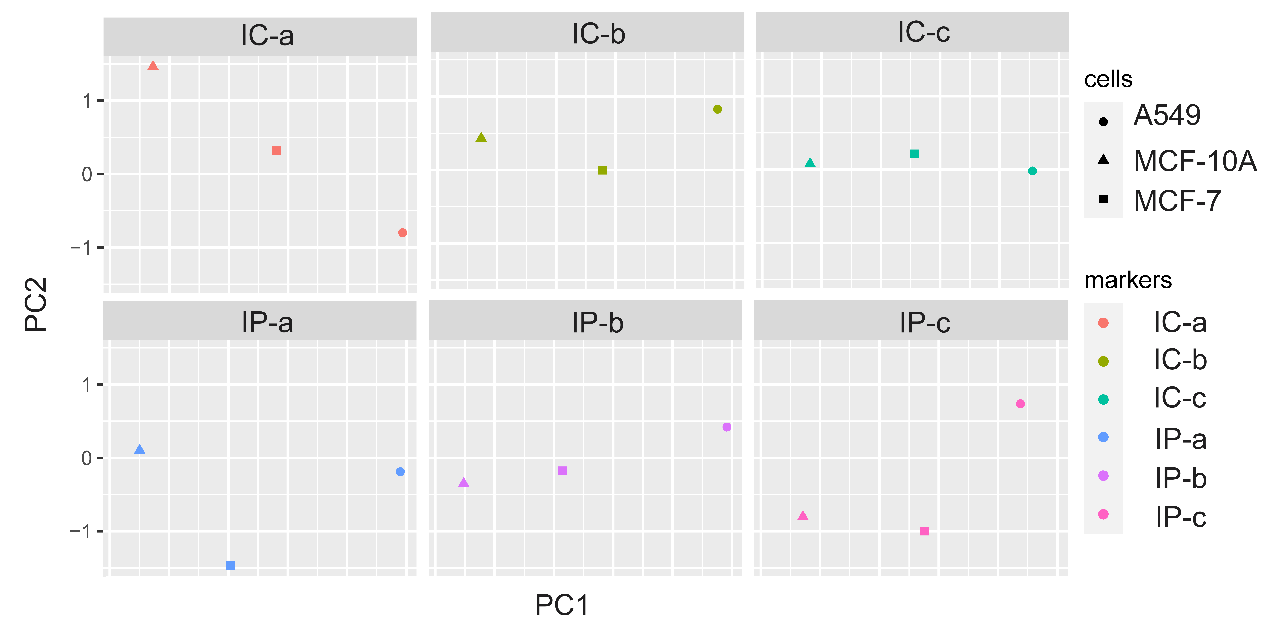


**Figure S19.** The independent analysis of individual concentration (IC) or individual proportion (IP) information for cells distinction. a) The IC and IP data were used as analysis information of principal component analysis (PCA) to obtain the corresponding cells differentiation view, respectively. The IC of individual analyte (i.e., IC-a, IC-b, and IC-c) and the IP toward let-7a, let-7b, and let-7c (i.e., IP-a, IP-b, and IP-c) outputted a visually distinguishable view.


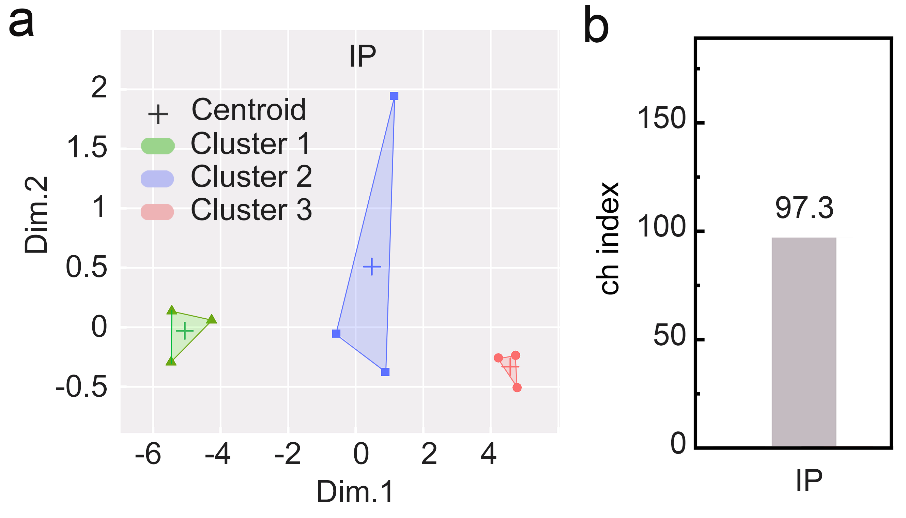


**Figure S20.** a) K-means clustering classification graph based on the PCA results and b) the ch index of K-means clustering classification graph at the IP level. The centroid corresponding to each cluster was marked with a plus sign in the corresponding color.

**Table S1**. Oligonucleotides used in this study.

| **Name** | **Sequences (5′-3′)** |
| --- | --- |
| capture probe | GAA TAG ATC TGG AAA TAG TTA C AC GAC CAC CCA GTG |
| CP | TTA GAT CAG-SH |
| B1 | TAA CAC TGG GTG GTC GTG CAG TGC TGC AGT AAT GAT  CAG ACT ATA CAA CCT ACT-SH |
| B2 | AGG CTT GC CCA GTA TGT GAC GAC CAC CCA GTG TTA  GAT CAG ACT ATA CAA CCT ACT-SH |
| B3 | CCT ACA GTT CTC ACT GTG ATA CTG GGC AAG CCT GAT  CAG ACT ATA CAA CCT ACT-SH |
| B4 | ATT ACT GCA GCA CTG GTG AGT GAG AAC TGT AGG TTT  TTTTTTTTTTTTTTTTTT AGT AGG TTG TAT AGT TTC GAT CAG |
| blocking | GAA ACT ATA CAA CCT ACT ACC TCA |
| let-7a | UGA GGU AGU AGG UUG UAU AGU U |
| let-7b | UGA GGU AGU AGG UUG UGU GGU U |
| let-7c | UGA GGU AGU AGG UUG UAU GGU U |
| let-7d | AGA GGU AGU AGG UUG CAU AGU U |
| let-7e | UGA GGU AGG AGG UUG UAU AGU |
| let-7f | UGA GGU AGU AGA UUG UAU AGU U |
| A1-1 | CTT TTT TTC TTC CTT ACC TCA |
| A2-1 | AAC TAT ACA ACC TAC AGG AAG ATA AAA AG |
| A1-2 | CTT TTT TTT CTT CCTT AC CTC A |
| A2-2 | AAC TAT ACA ACC TAC AGG AAG ATA AAA AAG |
| A1-3 | CTT TTT TTT TCT TCC TTA CCT CA |
| A2-3 | AAC TAT ACA ACC TAC AGG AAG AAT AAA AAA G |
| A1-4 | CTT TTT TTT TTC TTC CTT ACC TCA |
| A2-4 | AAC TAT ACA ACC TAC AGG AAG AAA TAA AAA AG |
| A1-5 | CTT TTT TTT TTT CTT CCT TAC CTC A |
| A2-5 | AAC TAT ACA ACC TAC AGG AAG AAA TAA AAA AAG |
| A2-b | AAC CAC ACA ACC TAC AGG AAG AAA /i6FAMdT/AA AAA AAA G |
| A2-c | AAC CAT ACA ACC TAC AGG AAG AAA /i6FAMdT/AA AAA AAA G |
| A1-B | CTT TTT TT/iBHQ1dT/ TTT CTT CCT TAC CTC A |
| A2-F | AAC TAT ACA ACC TAC AGG AAG AAA /i6FAMdT/AA AAA AAG |
| A1-s | C*T*T TTT TTT /iTAMdT/TT TCT TCC TTA CCT* C*A |
| A2-s | A*A*C TAT ACA ACC TAC AGG AAG AAA /i6FAMdT/AA AAA AA*A* G |
| anti-7a | A*A*C TAA ACA ACC TAC AAC CT*C* A |
| mimics | T*G*A GGT AGT AGG TTG TAT GG*T* T |
|  |  |

**Table S2**. The NGS- derived TFL and IC information.

| **let-7a** | | | **let-7b** | | | **let-7c** | | |
| --- | --- | --- | --- | --- | --- | --- | --- | --- |
|  | TFL | IC |  | TFL | IC |  | TFL | IC |
| MCF-7 | 621063 | 80005 | MCF-7 | 621063 | 21064 | MCF-7 | 621063 | 4681 |
| MCF-7 | 774035 | 98599 | MCF-7 | 774035 | 26116 | MCF-7 | 774035 | 5691 |
| MCF-7 | 594465 | 78182 | MCF-7 | 594465 | 20014 | MCF-7 | 594465 | 4528 |
| A549 | 403619 | 41373 | A549 | 403619 | 9606 | A549 | 403619 | 2642 |
| A549 | 667646 | 73427 | A549 | 667646 | 9086 | A549 | 667646 | 4281 |
| A549 | 624137 | 71080 | A549 | 624137 | 9417 | A549 | 624137 | 4135 |
| MCF-10A | 605047 | 162018 | MCF-10A | 605047 | 78606 | MCF-10A | 605047 | 5934 |
| MCF-10A | 537924 | 132121 | MCF-10A | 537924 | 69634 | MCF-10A | 537924 | 5219 |
| MCF-10A | 721286 | 156591 | MCF-10A | 721286 | 88652 | MCF-10A | 721286 | 6245 |

**Table S3**. Distances between each case and corresponding centroids in the IC view of K-means clustering.

| Case number | cluster | distance |
| --- | --- | --- |
| 1 | 1 | 1.00 |
| 2 | 1 | 0.49 |
| 3 | 1 | 1.15 |
| 4 | 2 | 0.64 |
| 5 | 2 | 0.33 |
| 6 | 2 | 0.87 |
| 7 | 3 | 0.19 |
| 8 | 3 | 0.25 |
| 9 | 3 | 0.41 |

**Table S4**. Distances between each case and corresponding centroids in the IP view of K-means clustering.

| Case number | cluster | distance |
| --- | --- | --- |
| 1 | 1 | 0.42 |
| 2 | 1 | 0.80 |
| 3 | 1 | 0.48 |
| 4 | 2 | 0.97 |
| 5 | 2 | 1.20 |
| 6 | 2 | 1.58 |
| 7 | 3 | 0.19 |
| 8 | 3 | 0.37 |
| 9 | 3 | 0.26 |

**Table S5**. Distances between each case and corresponding centroids in the IC-IP view of K-means clustering.

| Case number | cluster | distance |
| --- | --- | --- |
| 1 | 1 | 1.28 |
| 2 | 1 | 0.33 |
| 3 | 1 | 0.38 |
| 4 | 1 | 0.41 |
| 5 | 1 | 0.69 |
| 6 | 1 | 0.86 |
| 7 | 2 | 0.84 |
| 8 | 2 | 0.70 |
| 9 | 2 | 0.66 |
| 10 | 2 | 1.66 |
| 11 | 2 | 0.94 |
| 12 | 2 | 0.76 |
| 13 | 3 | 1.13 |
| 14 | 3 | .74 |
| 15 | 3 | 0.54 |
| 16 | 3 | 0.56 |
| 17 | 3 | 0.64 |
| 18 | 3 | 0.99 |

## Reference

[1] Kavakiotis, A. Alexiou, S. Tastsoglou, S. V. Ioannis, G. H. Artemis, *Nucleic Acids Res.*, **2022**, 50, D1055-D1061.

[2] Y. Gao, X. Y. Che n, T. R. Tian, S. J. Y. Gao, X. L. Zhang, Y. X. Yao, Y. F. Lin, X. X. Cai *Adv. Mater.,* **2022**, 34, 2201731. I.

[3] Y. Q. Xue, N. Liao, Y. Li, W. B. Liang, X. Yang, X. Zhong, Y. Zhuo, *Biosens. Bioelectron.,* **2022**, 217, 114713.

[4] E. S. Aslan, E. Aydin, *Naunyn-Schmiedeberg's Arch. Pharmacol.*, **2023**, 1-6.

[5] X. N. Lai, J. Li, L. B. Tang, W. T. Chen, L. Zhang, L. X. Xiong, *Int. J. Mol. Sci.*, **2020**, 21, 1193.

[6] Y. X. Ma, N. Shen, M. S. Wicha, M. Luo, *Cells*, **2021**, 10, 2415.
